# Disruption of Folate Metabolism Causes Poor Alignment and Spacing of Mouse Conceptuses for Multiple Generations

**DOI:** 10.3389/fcell.2021.723978

**Published:** 2021-12-10

**Authors:** Amy L. Wilkinson, Katerina Menelaou, Joanna Rakoczy, Xiu S. Tan, Erica D. Watson

**Affiliations:** Centre for Trophoblast Research, Department of Physiology, Development and Neuroscience, University of Cambridge, Cambridge, United Kingdom

**Keywords:** MTRR, trophoblast, decidua, grandparental effect, twinning, transgenerational epigenetic inheritance, conceptus misalignment

## Abstract

Abnormal uptake or metabolism of folate increases risk of human pregnancy complications, though the mechanism is unclear. Here, we explore how defective folate metabolism influences early development by analysing mice with the hypomorphic *Mtrr* ^*gt*^ mutation. MTRR is necessary for methyl group utilisation from folate metabolism, and the *Mtrr* ^*gt*^ allele disrupts this process. We show that the spectrum of phenotypes previously observed in *Mtrr* ^*gt/gt*^ conceptuses at embryonic day (E) 10.5 is apparent from E8.5 including developmental delay, congenital malformations, and placental phenotypes. Notably, we report misalignment of some *Mtrr* ^*gt*^ conceptuses within their implantation sites from E6.5. The degree of misorientation occurs across a continuum, with the most severe form visible upon gross dissection. Additionally, some *Mtrr* ^*gt/gt*^ conceptuses display twinning. Therefore, we implicate folate metabolism in blastocyst orientation and spacing at implantation. Skewed growth likely influences embryo development since developmental delay and heart malformations (but not defects in neural tube closure or trophoblast differentiation) associate with severe misalignment of *Mtrr* ^*gt/gt*^ conceptuses. Typically, the uterus is thought to guide conceptus orientation. To investigate a uterine effect of the *Mtrr* ^*gt*^ allele, we manipulate the maternal *Mtrr* genotype. Misaligned conceptuses were observed in litters of *Mtrr* ^*+/+*^, *Mtrr* ^*+/gt*^, and *Mtrr* ^*gt/gt*^ mothers. While progesterone and/or BMP2 signalling might be disrupted, normal decidual morphology, patterning, and blood perfusion are evident at E6.5 regardless of conceptus orientation. These observations argue against a post-implantation uterine defect as a cause of conceptus misalignment. Since litters of *Mtrr* ^*+/+*^ mothers display conceptus misalignment, a grandparental effect is explored. Multigenerational phenotype inheritance is characteristic of the *Mtrr* ^*gt*^ model, though the mechanism remains unclear. Genetic pedigree analysis reveals that severe conceptus skewing associates with the *Mtrr* genotype of either maternal grandparent. Moreover, the presence of conceptus skewing after embryo transfer into a control uterus indicates that misalignment is independent of the peri- and/or post-implantation uterus and instead is likely attributed to an embryonic mechanism that is epigenetically inherited. Overall, our data indicates that abnormal folate metabolism influences conceptus orientation over multiple generations with implications for subsequent development. This study casts light on the complex role of folate metabolism during development beyond a direct maternal effect.

## Introduction

Maternal folate deficiency in humans is associated with increased risk of congenital malformations (MRC Vitamin Study Research Group, 1991) and pregnancy complications, such as placental abruption, hemorrhage, and preeclampsia (Wen et al., 2008). These complications potentially lead to increased risk of spontaneous abortion, miscarriage, or pre-term birth (Bukowski et al., 2009). Yet, how folate metabolism functions in pregnancy establishment is not well understood. Folate, a vitamin that underpins one-carbon metabolism, is required for thymidine synthesis (Stover, 2004) and transfer of one-carbon methyl groups for cellular methylation reactions (Ducker and Rabinowitz, 2017). Therefore, folate is required for rapid cell division and epigenetic regulation of gene expression, which are both important for feto-placental development.

At implantation, human and mouse blastocysts embed into the uterine wall to improve fetal access to maternal resources. Mouse litters consist of 5–20 embryos that must be optimally spaced and oriented within the uterus for ideal growth. Spacing mechanisms are not well understood but likely involve mechanical forces generated by the uterus and cross talk between blastocysts and the uterine epithelium (Chen et al., 2013; Flores et al., 2020). The orientation of the developing mouse conceptus aligns with the antimesometrial-mesometrial axis of the decidual swelling, such that the placenta of each conceptus forms on the mesometrial side of the uterus (Smith, 1985). For normal alignment to occur, mouse blastocysts attach to the antimesometrial uterine epithelium *via* the mural trophectoderm (opposes the inner cell mass (ICM)) by embryonic day (E) 4.5 and invade into the decidualizing endometrium (Smith, 1985; Cross et al., 1994). In contrast, human blastocysts attach to the uterus *via* the polar trophectoderm (cells that contact the ICM) (Hertig et al., 1956). In mouse, the polar trophectoderm forms the ectoplacental cone (EPC) and extra-embryonic ectoderm, which together consist of progenitor populations of the mature placenta (Simmons and Cross, 2005; Watson and Cross, 2005). The base of the EPC abuts the chorion and the tip of the EPC extends upwards through the mesometrial decidua. The embryo forms below the chorion on the antimesometrial side of the implantation site. The maternal and embryonic factors required to establish normal conceptus orientation are not well studied, though it is likely determined by blastocyst orientation at implantation (Kirby et al., 1967; Red-Horse et al., 2004; Chen et al., 2013) and/or signals from the maternal decidua during post-implantation development (Alexander et al., 1996; Daikoku et al., 2011; Winterhager et al., 2013; Cha et al., 2014; Zhang et al., 2014). Furthermore, the extent to which deviation from normal conceptus alignment influences embryo and placenta development is not well understood.

Whether folate metabolism plays a role in implantation, spacing, and/or orientation remains unclear. Several mouse models of defective folate uptake, transport or metabolism suggest involvement of folate metabolism in early pregnancy. For instance, dietary folate deficiency in mice is associated with sub-fertility, increased embryonic lethality attributed to defects in decidualization (Gao et al., 2012; Geng et al., 2015), and low weight placentas with shallow trophoblast invasion (Pickell et al., 2009). During its metabolism, folate is converted to 5-methyltetrahydrofolate by methylenetetrahydrofolate reductase (MTHFR). Methionine synthase (MTR) then catalyzes the transfer of the methyl group from 5-methyltetrahydrofolate to homocysteine to produce methionine (Shane and Stokstad, 1985). Methionine is the precursor of S-adenosylmethionine, the methyl donor of all methylation reactions (Ducker and Rabinowitz, 2017). Importantly, methionine synthase reductase (MTRR) ensures progression of both folate and methionine cycles by continued activation of MTR through reductive methylation of its vitamin B_12_ co-factor (Yamada et al., 2006). Mutations in human *MTHFR, MTR,* and *MTRR* genes are linked to pregnancy complications (Ren and Wang, 2006; Furness et al., 2008; Kim et al., 2013; Wang et al., 2015), yet the mechanism of one-carbon metabolism in blastocyst spacing and conceptus orientation requires further study. Mutations in mouse genes associated with folate uptake or metabolism display gross placental phenotypes (e.g., *Slc19a1* ^*−/−*^, *Mthfr* ^*−/−*^ and *Mtrr* ^*gt/gt*^) or are yet-to-be assessed for defects beyond the embryo proper (e.g., *Folbp1* ^*−/−*^, *Folbp2* ^*−/−*^, *Slc46a1* ^*−/−*^, and *Mtr* ^*−/−*^) (Piedrahita et al., 1999; Swanson et al., 2001; Elmore et al., 2007; Gelineau-van Waes et al., 2008; Pickell et al., 2009; Salojin et al., 2011; Padmanabhan et al., 2013). Nevertheless, many of these mutations cause embryonic lethality by E10.5, a telltale sign of severe defects in placentation (Watson and Cross, 2005).

We previously reported the *Mtrr* ^*gt*^ knockdown mutation generated through a gene-trap (gt) insertion into the mouse *Mtrr* locus (Elmore et al., 2007; Padmanabhan et al., 2013). The *Mtrr* ^*gt*^ allele sufficiently disrupts one-carbon metabolism by significantly reducing MTR activity (Elmore et al., 2007). Indeed, *Mtrr* ^*gt/gt*^ mice exhibit characteristics similar to clinical indicators of human folate deficiency (Krishnaswamy and Madhavan Nair, 2001) including plasma hyperhomocysteinaemia, macrocytic anemia in adulthood, and a wide spectrum of developmental phenotypes at E10.5 including failure of neural tube closure (Elmore et al., 2007; Padmanabhan et al., 2013; Padmanabhan et al., 2018). Therefore, the *Mtrr* ^*gt*^ mutation is a robust model to assess the effects of abnormal folate metabolism. Besides neural tube closure defects, other developmental phenotypes observed in *Mtrr* ^*gt/gt*^ conceptuses at E10.5 are growth restriction, developmental delay, cardiac malformations, and placenta phenotypes (Padmanabhan et al., 2013). While the molecular mechanism and developmental timing of these phenotypes is currently unclear, alterations in genome-wide DNA methylation associated with gene misexpression are apparent in *Mtrr* ^*gt/gt*^ conceptuses at E10.5 (Padmanabhan et al., 2013; Bertozzi et al., 2021; Blake et al., 2021). This result implicates an epigenetic mechanism.

Interestingly, the *Mtrr* ^*gt*^ mouse line also displays transgenerational epigenetic inheritance of developmental phenotypes (Padmanabhan et al., 2013). Transgenerational epigenetic inheritance is a non-conventional mode of inheritance caused by environmental stressors and/or metabolic disruption present only in the F0 generation that results in phenotypes at least up to the F3 generation (Blake and Watson, 2016). The mechanism of mammalian transgenerational epigenetic inheritance is not well understood. It is hypothesized that an epigenetic factor(s) is inherited *via* the germline in a manner that is independent of DNA mutation (Blake and Watson, 2016), yet it is difficult to tease apart the effects of the uterine environment from those intrinsic to the germline. In the case of the *Mtrr* ^*gt*^ mouse line, an *Mtrr* ^*gt*^ allele in either maternal grandparent (i.e., the F0 generation) is sufficient to disrupt germ cell DNA methylation (Blake et al., 2021) and cause growth defects and congenital malformations at E10.5 in the wildtype grandprogeny, in some cases up to the F4 wildtype generation (Padmanabhan et al., 2013). This phenomenon occurs when the F1 generation and all subsequent generations are wildtype for the *Mtrr* gene and display normal folate metabolism (Padmanabhan et al., 2013; Padmanabhan et al., 2018). While robust genetic pedigree analysis and embryo transfer experiments showed that congenital malformations occurred independent of the uterine environment (Padmanabhan et al., 2013), the specific mechanism of transgenerational epigenetic inheritance is yet-to-be determined in this and other models.

The overall study aim is to understand how defective folate metabolism due to the *Mtrr* ^*gt*^ mutation influences early development and the uterine- and embryo-specific effects involved. Notably, we observe a spectrum of conceptus misalignment from E6.5. The most severe form of this phenotype is visible at gross dissection as “eccentric placenta development” and associates with specific embryonic defects at midgestation. This finding has implications for the origin of some congenital malformations associated with folate deficiency. Misalignment occurred alongside twinning, which links folate metabolism to blastocyst orientation and spacing at implantation. To better understand the mechanisms behind conceptus misalignment, we investigate a maternal effect of the *Mtrr* ^*gt*^ allele. Data collected from histological analyses, highly controlled genetic pedigrees, and embryo transfer experiments reveal that severe conceptus misalignment occurs independent of the peri- and/or post-implantation uterus. This result differs from other mouse models displaying skewed growth. Instead, our data supports an embryo-specific defect as a cause of conceptus misorientation that is epigenetically inherited from either *Mtrr* ^*+/gt*^ maternal grandparent. Overall, we propose that defective folate metabolism disrupts blastocyst orientation and spacing over multiple generations, with implications for subsequent feto-placental development and a mechanism beyond a direct maternal effect.

## Materials and Methods

### Ethics

All experiments were performed in accordance with UK Home Office regulations under the Animals (Scientific Procedures) Act 1986 Amendment Regulations 2012 and underwent review by the University of Cambridge Animal Welfare and Ethical Review Body.

### Mice

The *Mtrr* ^*gt*^ mouse line was generated as previously described (Padmanabhan et al., 2013). Briefly, a gene-trap (gt) vector was inserted into intron 9 of the *Mtrr* gene. Upon germline transmission, the *Mtrr* ^*gt*^ allele was backcrossed to C57Bl/6J mice for eight generations (Padmanabhan et al., 2013). C57Bl/6J mice (The Jackson Laboratory), which are wildtype for the *Mtrr* gene, were used as controls and were bred in house separately from the *Mtrr* ^*gt*^ mouse line. All mice were housed in a temperature- and humidity-controlled environment with a 12-h light/dark cycle, and fed a standard chow (Rodent No. 3 chow; Special Diet Services, Essex, United Kingdom) *ad libitum* from weaning (includes folic acid at 2.99 mg/kg of diet, methionine at 0.37%, choline at 1422.4 mg/kg of diet, and vitamin B_12_ at 19.2 μg/kg of diet). *Mtrr* ^*gt/gt*^ conceptuses were derived from *Mtrr* ^*gt/gt*^ intercrosses unless otherwise stated. For assessment of maternal effect, *Mtrr* ^*+/+*^, *Mtrr* ^*+/gt*^ and *Mtrr* ^*gt/gt*^ female mice derived from *Mtrr* ^*+/gt*^ intercrosses were mated with C57Bl/6J males, and the resulting litters were dissected at E6.5. For assessment of maternal grandparental effect, F0 *Mtrr* ^*+/gt*^ male or female mice were crossed with a C57Bl/6J mate. Their F1 *Mtrr* ^*+/+*^ daughters were mated with C57Bl/6J males to generate F2 *Mtrr* ^*+/+*^ progeny for dissection at E10.5 or for littering out. F2 *Mtrr* ^*+/+*^ females were mated with C57Bl/6J males to generate F3 *Mtrr* ^*+/+*^ progeny for dissection at E10.5 or littering out. F3 *Mtrr* ^*+/+*^ females were mated with C57Bl/6J males to generate F4 *Mtrr* ^*+/+*^ conceptuses for dissection at E10.5.

### Genotyping

DNA samples were obtained from ear tissue or yolk sac for polymerase chain reaction (PCR) genotyping of the *Mtrr* ^*gt*^ allele or sex as reported previously (Padmanabhan et al., 2013; Tunster, 2017).

### Dissections and Phenotyping

Noon of the day that the vaginal plug was detected was considered E0.5. Pregnant female mice were euthanized *via* cervical dislocation. Dissections were performed in cold 1x phosphate buffered saline (PBS). Each conceptus was individually scored for phenotypes (*see* below). Whole implantation sites at E6.5, or placentas and embryos separately at E8.5, E10.5, E14.5, and E18.5 were weighed, photographed, and either snap frozen in liquid nitrogen for molecular analysis or fixed in 10% natural-buffered formalin (Cat. No. HT501128; Sigma-Aldrich) at 4°C overnight and paraffin embedded for sectioning using standard protocols.

A rigorous phenotyping regime was performed as previously described (Padmanabhan et al., 2013). Briefly, conceptuses were defined as “phenotypically normal” if they displayed 6–12 somite pairs at E8.5 and 30–40 somite pairs at E10.5 (according to e-Mouse Atlas Project; http://www.emouseatlas.org) and displayed no growth defect or gross abnormalities. For analysis at E10.5 only, an embryo was growth restricted if its crown-rump length was less than two standard deviations from the control C57Bl/6J mean crown-rump length. Growth restricted embryos also displayed normal somite pair counts and no other phenotype. At E8.5 and E10.5, developmentally delayed conceptuses exhibited <6 somite pairs or <30 somite pairs, respectively, but were otherwise normal for the developmental stage indicated by the somite pair counts. Severely affected conceptuses displayed one or more abnormality with appreciation for the developmental stage (e.g., somite pair count), as some of these embryos were also developmentally delayed. Abnormalities included: *1*) delay neural tube closure whereby the length of the neural plate that adhered to form a tube was shorter than expected for the somite stage at E8.5, or failure of neural tube closure within the cranial or spinal cord region at E10.5, *2*) heart defects (E10.5 only) including heart looping defect, pericardial edema, or hemorrhage, *3*) placenta defects including failure of chorioallantoic attachment or eccentrically located placentas, or *4*) “twinning” whereby two or more conceptuses shared a single implantation site. An implantation site that consisted of a decidual swelling with an ill-defined mass of fetal-derived cells was categorized as a resorption and considered dead. Conceptuses were allocated a phenotypic category based on their most severe phenotype and were only counted once.

### Embryo Transfer

Embryo transfer experiments were performed and analyzed as previously described in detail (Padmanabhan et al., 2013). Briefly, using M2 media (Cat. No. M7167; Sigma-Aldrich), pre-implantation wildtype embryos were flushed at E3.25 from the oviducts and uteri of *Mtrr* ^*+/+*^ females (derived from one *Mtrr* ^*+/gt*^ parent) mated with C57Bl/6J males. Donor females were not superovulated. After a brief embryo culture period to prepare the recipient females, embryos were surgically transferred into the uteri of E2.5 pseudopregnant [C57Bl/6J × DBA/2] F1 hybrid (B6D2F1) females (Charles River). Control C57Bl/6J embryos were similarly transferred. Donor and recipient females were 7–10 weeks old. Embryos were transferred regardless of appearance and litters were never pooled. Transferred embryos were dissected at E10.5, the timing of which corresponded to the staging of the recipient female (Ueda et al., 2003), and scored for phenotypes as above.

### Immunohistochemistry

Whole implantation sites or placentas were carefully oriented in paraffin for transverse sectioning at 5 μm for conceptuses at E6.5 and 7 μm for conceptuses at E8.5. Tissue sections that were centrally located were selected for analysis, and deparaffinized in xylene (Cat. No. IS8126; Thermo Fisher Scientific) before rehydration in descending concentrations of ethanol. Hematoxylin (Cat No. MHS32; Sigma-Aldrich) and eosin (Cat No. 861006; Sigma-Aldrich) staining of histological sections was performed using standard protocols. For immunohistochemistry staining, endogenous peroxidase activity was quenched using 3% H_2_O_2_ (Cat No. H/1750/15; Thermo Fisher Scientific). Antigen retrieval was performed by treatment of tissue sections with porcine trypsin tablets (Cat No. T7168-20TAB; Sigma-Aldrich) for 10 min at room temperature. Tissue sections were first washed with 1× PBST (1× PBS with 20% Tween20) and then in blocking serum (5% serum, Cat. No. D9663; Sigma-Aldrich), 1% bovine serum albumin (BSA, Cat. No. A4503; Sigma-Aldrich) in 1× PBST for 1 h. Sections were exposed to primary antibodies diluted in blocking serum overnight at 4°C. The primary antibodies and dilutions used were: 1:300 rabbit polyclonal anti-COX2 (Cat. No. ab15191, RRID:AB_2085144; abcam, Cambridge, United Kingdom), 1:100 rabbit anti-MTRR (Cat. No. 26944-1-AP, RRID:AB_2880694; Proteintech Europe, Manchester, United Kingdom), and 1:150 rabbit anti-PGR (Cat. No. ab63605, RRID:AB_1142326; abcam). After thorough washing in 1× PBST, tissue sections were incubated for 1 h at room temperature with donkey anti-rabbit IgG polyclonal antibody conjugated with horseradish peroxidase (Cat. No. ab6802, RRID:AB_955445; abcam) diluted to 1:300 in blocking serum. Peroxidase substrate reactions were conducted with 3,3′-diaminobenzidine (DAB) chromagen substrate kit according to the manufacturer’s instructions (Cat. No. ab64238; abcam). Tissue sections were counterstained with haematoxylin and coverslip mounted using DPX mountant (Cat. No. 360294; VWR).

### *In Situ* Hybridization

*In situ* hybridisation probes were generated with gene-specific primer sets (Supplementary Table S1) that contained T7 RNA polymerase promoter sequence (TAA​TAC​GAC​TCA​CTA​TAG​GG) attached to each reverse primer and T3 RNA polymerase promoter sequence (AAT​TAA​CCC​TCA​CTA​AAG​GG) attached to each forward primer (Outhwaite et al., 2019). Probe synthesis of digoxigenin (DIG)-labelled anti-sense and sense probes was performed *via* PCR using DIG RNA labelling Mix (Cat. No. 11277073910; Roche, Welwyn Garden City, United Kingdom) according to the manufacturer’s instructions. The *in situ* hybridization protocol was performed as previously described (Simmons et al., 2008) with the following alteration: paraffin-embedded tissue sections were used and thus, an initial dewaxing/rehydration step was added.

### Quantitative Reverse Transcription PCR (RT-qPCR)

RNA was extracted from whole implantation sites at E6.5 using Trizol (Cat. No. T9424; Sigma-Aldrich) according to the manufacturer’s instructions. cDNA was synthesised using RevertAid H Minus reverse transcriptase (Cat. No. EP0452; Thermo Fisher Scientific) and random hexamer primer (Cat. No. SO142; Thermo Fisher Scientific) using 1–2 μg of RNA in a 20-μl reaction according to manufacturer’s instructions. PCR amplification was performed using MESA Green qPCR MasterMix for SYBR assay (Cat. No. RT-SY2X-03+WOUN; Eurogentec, Ltd.) on a DNA Engine Opticon2 thermocycler (Bio-Rad). Experiments were conducted with technical duplicates or triplicates and using at least four biological replicates. Transcript levels were normalised to *Polr2a* (Solano et al., 2016) and analyzed using the ΔΔCt method (Livak and Schmittgen, 2001). Transcript levels in C57Bl/6J tissue were controls (normalized to 1). For primer sequences, *see* Supplementary Table S1.

### Statistics

Statistical analyses were performed using GraphPad Prism 8 software (La Jolla, CA, United States). Parametric data was analyzed by unpaired *t* test or one-way ANOVA. Non-parametric data was analyzed by Mann Whitney test. Correlations were analyzed by linear regression or Fisher’s exact test. *p* < 0.05 was considered significant.

### Software and Imaging

Primer sequences were designed using the NCBI primer design tool (Ye et al., 2012). Conceptuses were dissected using a Zeiss SteReo Discovery V8 microscope and photographed with an AxioCam MRc5 camera and AxioVision 4.7.2 software (Carl Zeiss). Tissue sections were scanned using a NanoZoomer (Hamamatsu Photonics, United Kingdom) and viewed using NDP Scan 2.7 software (Hamamatsu Photonics). Images were analyzed using ImageJ software (NIH, Bethesda, MD, United States). Graphs were generated using Graphpad Prism 8 software.

## Results

### *Mtrr* ^*gt/gt*^ Conceptuses Exhibit Developmental Phenotypes at E8.5

**FIGURE 1:**
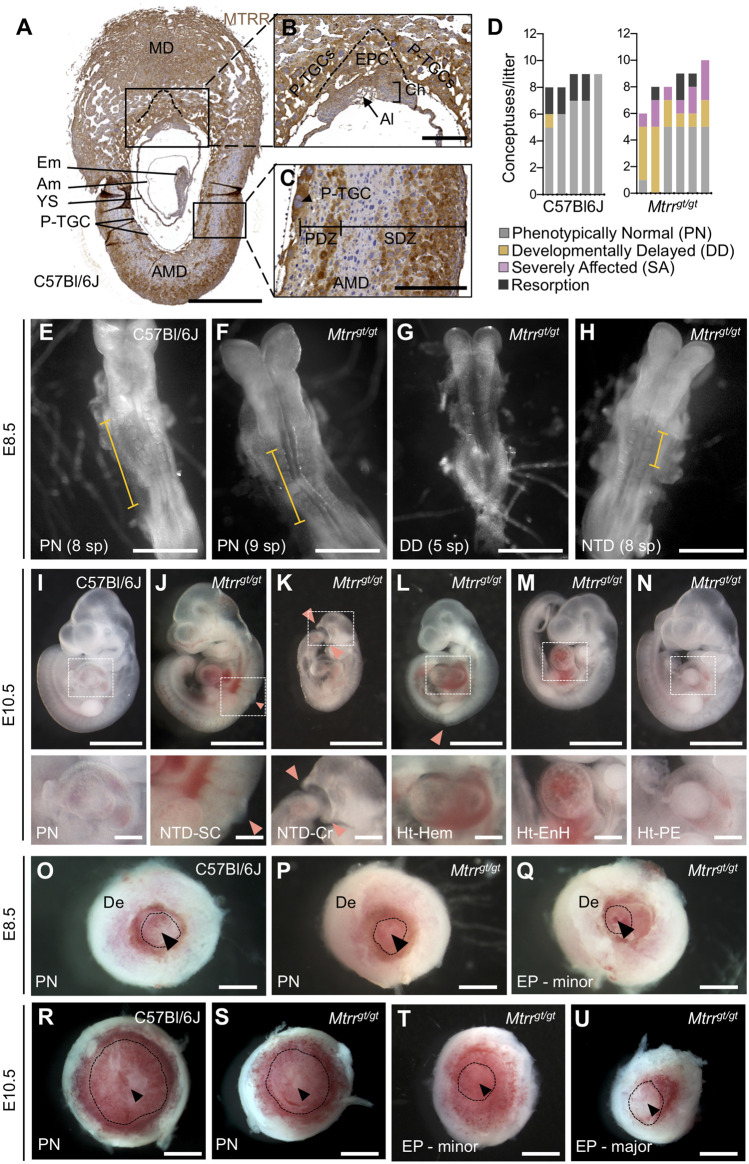
Spectrum of phenotypes observed in *Mtrr* ^*gt/gt*^ mouse conceptuses at E8.5 and E10.5. **(A**–**C)** MTRR protein expression (brown) in a wildtype C57Bl/6J implantation site at E8.5. DNA, blue. Boxed regions in **(A)** shown at higher magnification in **(B**,**C)**. *N* = 5 implantation sites, three technical replicates. Dashed line, boundary between EPC and P-TGCs. **(D)** Graph depicting the frequency of phenotypes per litter at E8.5 from C57Bl/6J control crosses and *Mtrr* ^*gt/gt*^ crosses. Each bar represents one litter. Grey, phenotypically normal; black, resorption; yellow, developmental delay (<6 somite pairs); pink, severely affected. *See* also Table 1. **(E**–**H)** Images of **(E)** C57Bl/6J and **(F**–**H)** *Mtrr* ^*gt/gt*^ embryos captured at E8.5 from **(E**,**F)** phenotypically normal (PN), **(G)** developmentally delayed (DD), or **(H)** severely affected (SA) conceptuses. The *Mtrr* ^*gt/gt*^ embryo in **(H)** displays a neural tube with delayed closure. Yellow bar indicates the length of neural plate that has closed into a tube. **(I**–**N)** Images of **(I)** phenotypically normal (PN) C57Bl/6J embryos at E10.5 and **(J**–**N)** severely affected *Mtrr* ^*gt/gt*^ embryos at E10.5 displaying **(J–L)** neural tube closure defects (pink arrowhead) in the **(J**,**L)** spinal cord region or **(K)** cranial region, or **(L**–**N)** heart malformations including **(L)** hemorrhage, **(M)** pericardial edema, or **(N)** an enlarged heart. Box depicts region of higher magnification directly below. **(O**–**U)** Images of placentas from **(O**,**R)** C57Bl/6J and **(P**,**Q**,**S**–**U)** *Mtrr* ^*gt/gt*^ conceptuses **(O**–**Q)** at E8.5 and **(R**–**U)** at E10.5 that were phenotypically normal (PN) or eccentrically located (EP). Dashed line, approximate outline of chorion. Black arrowhead, region of allantois attachment. Scale bars: **(A**,**O**–**U)** 1 mm, **(B**,**C)** 250 μm, **(E**–**H)**: 500 μm, **(I**–**N)** low magnification, 500 μm, high magnification, 100 μm. Al, allantois; Am, amnion; AMD, antimesometrial decidua; Ch, chorion; Cr, cranial; De, decidua; Em, embryo proper; EnH, enlarged heart; EP, eccentric placenta (severe conceptus misalignment); EPC, ectoplacental cone; Hem, hemorrhage; Ht, heart defect; MD, mesometrial decidua; NTD, neural tube defect; PDZ, primary decidual zone; PE, pericardial edema; P-TGCs, parietal trophoblast giant cells; SC, spinal cord; SDZ, secondary decidual zone; sp, somite pairs; YS, yolk sac.

To better understand the effects of the *Mtrr* ^*gt/gt*^ mutation on early mouse development, we first determined where MTRR enzyme functions in the conceptus by performing immunohistochemistry on histological sections of whole C57Bl/6J implantation sites at E8.5. MTRR protein was widely expressed in wildtype embryonic and extra-embryonic cell types including the yolk sac, amnion, allantois, and trophoblast progenitors (i.e., the chorion and EPC) at E8.5 (Figures 1A–C). This expression pattern was consistent with other folate metabolic enzymes and transporters at E8.5 (Cherukad et al., 2012). While expressed throughout the mesometrial and lateral decidua (Figure 1A), the antimesometrial decidua displayed striped MTRR expression in the primary and secondary decidual zones at E8.5 (Figure 1C) suggesting regionalized expression in the decidua. Overall, the broad expression of MTRR protein in wildtype conceptuses at E8.5 suggested a potential mechanistic role of folate metabolism in early feto-placental development and decidualization. *Mtrr* ^*gt/gt*^ embryos and placentas at midgestation display a substantial decrease in wildtype *Mtrr* transcript levels (6–24% of control levels) as determined by RT-qPCR (Padmanabhan et al., 2013), emphasizing the knockdown effect of the *Mtrr* ^*gt*^ allele.

Next, to explore the extent of developmental disruption caused by the *Mtrr* ^*gt/gt*^ mutation before E10.5, rigorous phenotype assessment of *Mtrr* ^*gt/gt*^ conceptuses was performed at E8.5. *Mtrr* ^*gt/gt*^ conceptuses derived from *Mtrr* ^*gt/gt*^ intercrosses were scored for gross abnormalities (*N* = 50 conceptuses from six litters). C57Bl/6J conceptuses, which are genetically wildtype for *Mtrr* and bred separately from the *Mtrr* ^*gt*^ mouse line, were used as controls (*N* = 43 conceptuses from five litters). This is because the *Mtrr* ^*gt*^ allele was backcrossed into the C57Bl/6J mouse strain and *Mtrr* ^*+/+*^ conceptuses derived from *Mtrr* ^*+/gt*^ intercrosses display multigenerational inheritance of developmental phenotypes (Padmanabhan et al., 2013). Similar to E10.5 (Padmanabhan et al., 2013), *Mtrr* ^*gt/gt*^ litter sizes and resorption rates were consistent with controls at E8.5 (Figure 1D; Table 1). Allocation to a phenotypic category depended upon the most severe phenotype exhibited by each conceptus (*see* methods). The majority of C57Bl/6J conceptuses at E8.5 were phenotypically normal (79.1% of conceptuses), displaying 6–12 somite pairs and no other obvious abnormalities (Figures 1D,E; Table 1). Nearly all other C57Bl/6J conceptuses were resorbed (18.6% of conceptuses; Figure 1D; Table 1), appearing as decidual swellings with an ill-defined mass of fetal-derived cells. In contrast, only 42.0% of *Mtrr* ^*gt/gt*^ conceptuses at E8.5 were phenotypically normal upon gross inspection (Figures 1D,F; Table 1; *p* = 0.009). The remaining *Mtrr* ^*gt/gt*^ conceptuses were either resorbed (8.0% of conceptuses; Figure 1D; Table 1), or exhibited one or more developmental phenotype (Figures 1D,G,H; Table 1). These phenotypes included developmental delay, defined as embryos with <6 somite pairs yet otherwise normal for the developmental stage (30.0% of conceptuses; *p* = 0.032; Figures 1D,G; Table 1), or severe abnormalities (20.0% of conceptuses; *p* = 0.0013; Figures 1D,H; Table 1). Defects that were considered severe were delay of neural tube closure (10% of conceptuses; Table 1) whereby the length of the neural plate that formed a tube was shorter than expected based on somite pair count at E8.5 (Figure 1H) and might lead to failure of neural tube closure by E10.5 (Figures 1I–L), and/or placenta phenotypes whereby chorioallantoic attachment was absent (4% of conceptuses) or the placenta was eccentrically located (12% of conceptuses; Figures 1P,Q; Table 1). In contrast, C57Bl/6J conceptuses at E8.5 infrequently displayed developmental delay (2.3% of conceptuses; Table 1) and severe abnormalities were undetected (Figures 1D,I,O; Table 1). The phenotypes in *Mtrr* ^*gt/gt*^ conceptuses at E8.5 were similar to those observed at E10.5 (Figures 1J,K), though gross heart defects including hemorrhage, enlargement, and pericardial edema observed at E10.5 (Figures 1L–N) (Padmanabhan et al., 2013) were not observed at E8.5. A more detailed assessment of the heart at E8.5 is required in the future. Overall, some developmental phenotypes caused by the *Mtrr* ^*gt*^ mutation likely originate prior to or at E8.5 (e.g., neural tube closure defects and placenta defects) while others do not become apparent until after E8.5 (e.g., heart defects).

**TABLE 1:** Frequency of developmental phenotypes in *Mtrr* ^*gt/gt*^ conceptuses at E8.5.

Embryonic genotype	C57Bl/6J	*Mtrr* ^*gt/gt*^
No. of conceptuses (No. of litters)	43 (5)	50 (6)
No. of conceptuses/litter	8.6 ± 0.2	8.3 ± 0.6
Phenotypically Normal [6–12 somite pairs]	6.8 ± 0.7 (79.1%)	3.5 ± 1.0^a^ (42.0%)
Developmentally Delay [<6 somite pairs]	0.2 ± 0.2 (2.3%)	2.5 ± 0.7^b^ (30.0%)
Severe abnormalities^c^	0.0 ± 0.0 (0.0%)	1.6 ± 0.3^a^ (20.0%)
Eccentric placenta location	0.0 ± 0.0 (0.0%)	1.0 ± 0.5 (12.0%)
No chorioallantoic attachment	0.0 ± 0.0 (0.0%)	0.3 ± 0.2 (4.0%)
Neural tube closure defects	0.0 ± 0.0 (0.0%)	0.8 ± 0.3^b^ (10.0%)
Resorption	1.6 ± 0.4 (18.6%)	0.7 ± 0.3 (8.0%)

Data displayed as mean incidence per litter ± se. Percentage of all conceptuses assessed in brackets.

a *p* < 0.01.

b *p* < 0.05.

c Conceptuses that displayed severe abnormalities were often developmentally delayed. These conceptuses were only counted once and were attributed to the most severe phenotype category. Eccentric placenta location, no chorioallantoic attachment, and neural tube closure defects were subcategories of severe abnormalities.

### A Low Frequency of Twinning Occurs in *Mtrr* ^*gt/gt*^ Conceptuses Throughout Gestation

**FIGURE 2:**
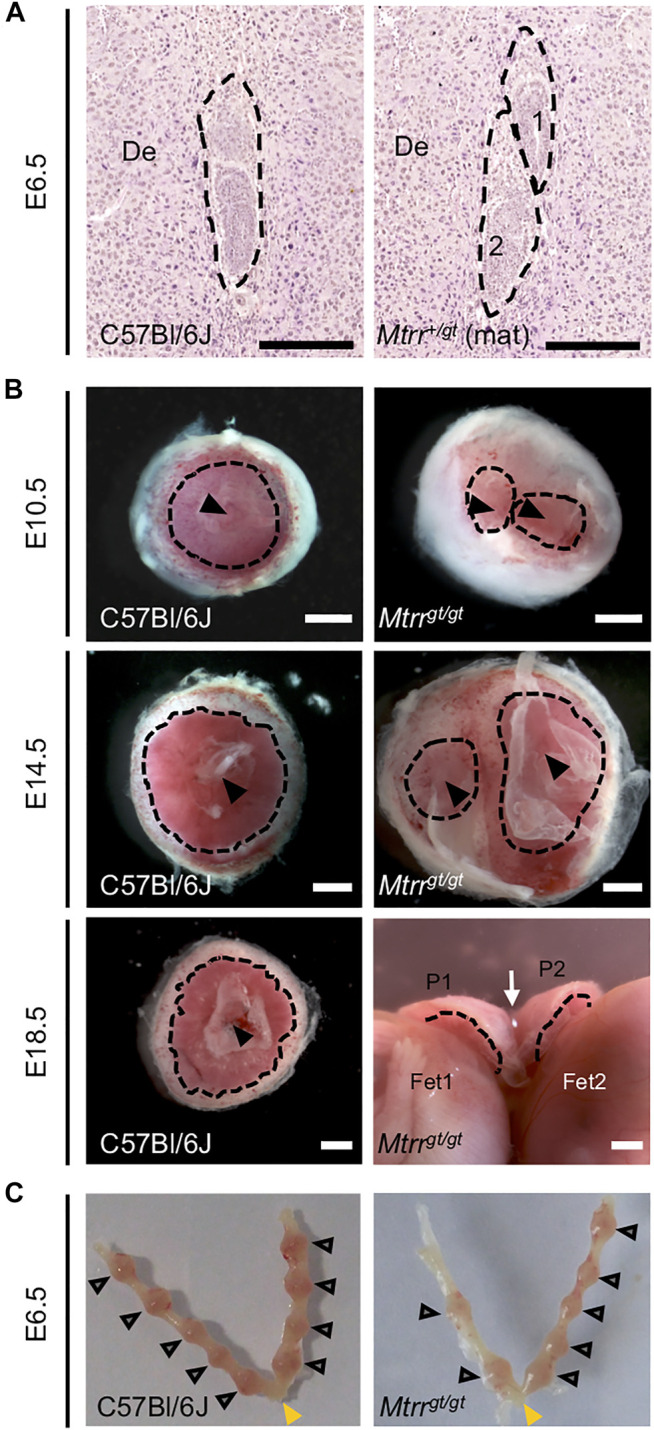
Twinning observed in *Mtrr* ^*gt/gt*^ mouse conceptuses across gestation. **(A**,**B)** Images depicting twinning at specific stages of development. **(A)** Histological sections of a C57Bl/6J implantation site at E6.5 with a single conceptus (dashed outline, **left-hand image**) and of an implantation site at E6.5 with two conceptuses (dashed outlines, **right-hand image**) derived from *Mtrr* ^*+/gt*^ mother (*Mtrr* ^*+/gt*^ mat) and C57Bl/6J father. Scale bar, 250 μm. **(B)** Whole placentas from singleton C57Bl/6J conceptuses (**left-hand images**) and twinned *Mtrr* ^*gt/gt*^ conceptuses (**right-hand images**) at E10.5, E14.5 and E18.5. Placentas viewed from the fetal-facing chorionic plate. Black dotted line, approximate outline of labryinth; black arrowhead, allantois attachment site; white arrow indicates where the placentas at E18.5 are fused. De, decidua; P, placenta; Fet, fetus. Scale bars, 1 mm. **(C)** Dissected uteri at E6.5 showing normal conceptus spacing in C57Bl/6J and *Mtrr* ^*gt/gt*^ litters. Grey arrowheads, individual implantation sites. Yellow arrowhead, cervix.

During the phenotypic assessment of *Mtrr* ^*gt*/gt^ conceptuses, we also observed a low frequency of dizygotic twinning that was apparent from E6.5 to E18.5 (0.7–2.3% of implantation sites, *N* = 118–308 sites (stage specific)) (Figures 2A,B). Twinning occurred when at least two conceptuses occupied a single decidual swelling. This phenotype was never detected in C57Bl/6J controls (*N* = 314 conceptuses). The occurrence of male and female embryos together in a single implantation site indicated dizygosity and suggested that twinning in the *Mtrr* ^*gt*^ mouse line likely resulted from poor blastocyst spacing at implantation. However, similar to controls, *Mtrr* ^*gt/gt*^ implantation sites were generally evenly spaced along the length of *Mtrr* ^*gt/gt*^ uteri (Figure 2C) rather than clustered together as is observed when there is a major uterine defect (Lu et al., 2013; Flores et al., 2020). Also, no more than one twinned implantation site was usually observed per *Mtrr* ^*gt/gt*^ litter (this study; Padmanabhan et al., 2013) indicating incomplete phenotype penetrance. Therefore, a uterine-specific defect was unlikely. Instead, we hypothesise that twinning caused by the *Mtrr* ^*gt/gt*^ mutation reflected an embryo-specific defect.

### *Mtrr* ^*gt/gt*^ Mutation Causes Skewed Orientation of the Entire Conceptus

Given the high frequency of gross placental phenotypes identified in *Mtrr* ^*gt/gt*^ conceptuses at E8.5 and E10.5 (Padmanabhan et al., 2013), the placenta was assessed further. No placental phenotypes were detected in C57Bl/6J control conceptuses at E8.5 or E10.5 (Table 1; Figures 1O,R) (Padmanabhan et al., 2013). However, *Mtrr* ^*gt/gt*^ conceptuses showed two distinct gross placental phenotypes. The first defect was failure of the allantois to contact and attach to the chorion (E8.5: 4.0% of conceptuses (Table 1); E10.5: 0.3% of conceptuses (Padmanabhan et al., 2013)), which prevents subsequent placental formation and causes embryonic lethality (Cross et al., 2003). The cause of this chorioallantoic attachment defect was unclear and it was not considered further here. The second phenotype was an eccentric placenta whereby the region of allantoic attachment appeared askew within the implantation site (E8.5: 12.0% of conceptuses (Table 1; Figures 1P,Q); E10.5: 5.8% of conceptuses (Figures 1S–U) (Padmanabhan et al., 2013)). Declining phenotypic frequencies during gestation suggested that the phenotype normalized or some *Mtrr* ^*gt/gt*^ conceptuses with these placental defects underwent lethality between E8.5 and E10.5.

**FIGURE 3:**
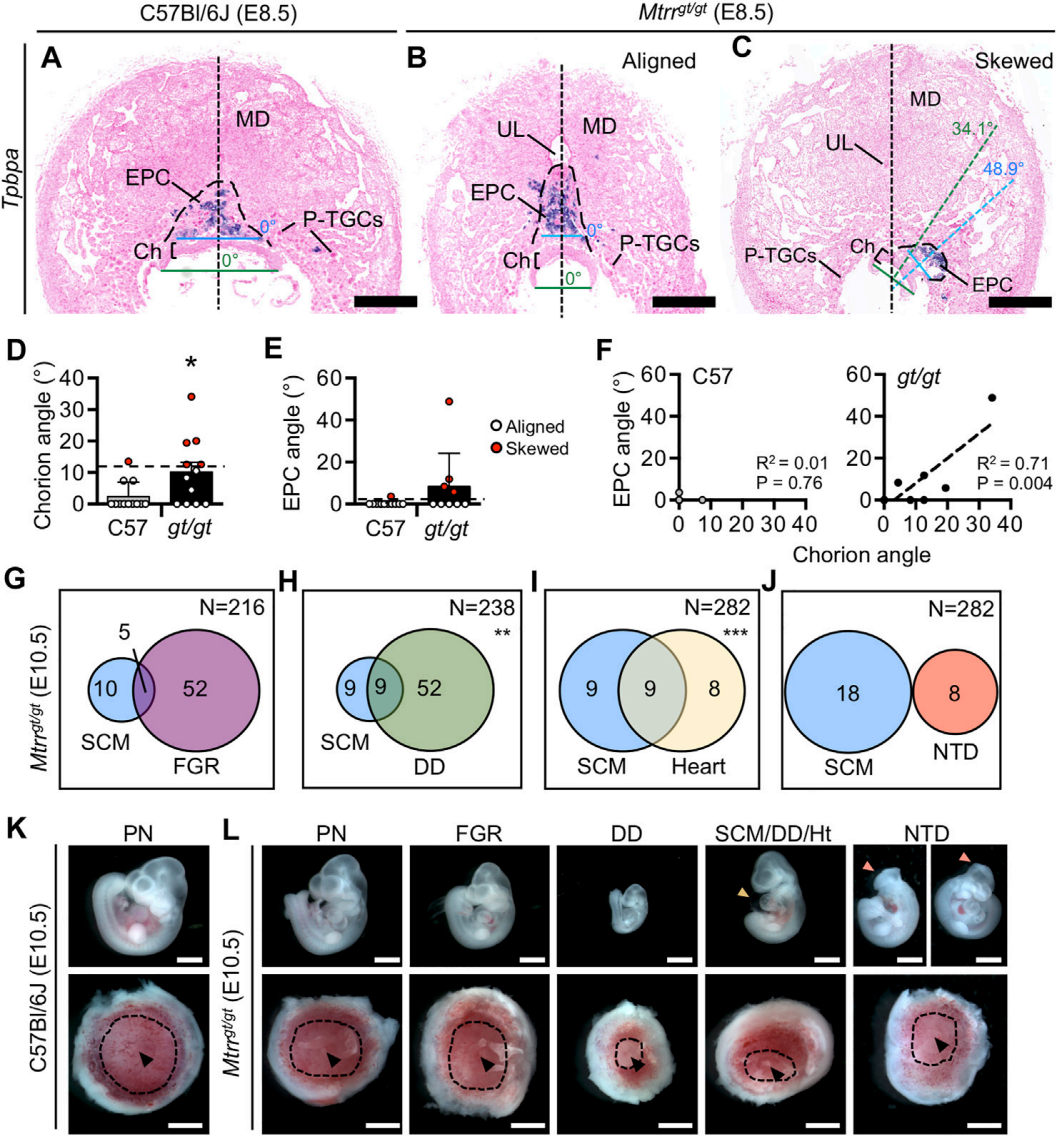
*Mtrr* ^*gt/gt*^ mouse conceptuses at E8.5 demonstrate misaligned orientation with incomplete penetrance. **(A**–**C)** Histological sections of placentas at E8.5 from **(A)** a C57Bl/6J conceptus and **(B**,**C)** *Mtrr* ^*gt/gt*^ conceptuses with **(B)** aligned or **(C)** skewed orientation. Ectoplacental cone (EPC, outer boundary indicated by dashed line) is depicted by *Tpbpa* mRNA expression (purple) as determined *via in situ* hybridization. Black dotted line, bisection of placenta. Green dotted line, bisection of the chorion. Blue dotted line, bisection of the EPC. The angle between the line bisecting the placenta and the chorion or EPC is shown. **(D**,**E)** Data showing the average (mean ± s.d.) **(D)** chorion angle or **(E)** EPC angle in C57Bl/6J (C57) controls and *Mtrr* ^*gt/gt*^ (*gt/gt*) conceptuses at E8.5 (*N* = 9–12 conceptuses/group). Conceptuses with chorion or EPC angles >2 s.d. above the control mean (black dashed line [chorion = 11.4°; EPC = 2.4°]) were defined as skewed and represented as red data points. Two-tailed Mann-Whitney test, *p = 0.028. **(F)** Linear regression analysis between chorion and EPC angles for C57Bl/6 conceptuses (C57; *N* = 10 conceptuses) and *Mtrr* ^*gt/gt*^ conceptuses (*gt/gt*; *N* = 9 conceptuses). **(G**–**J)** Venn diagrams depicting overlap between severe conceptus misalignment (SCM) in *Mtrr* ^*gt/gt*^ conceptuses at E10.5 with embryonic phenotypes including **(G)** fetal growth restriction, **(H)** developmental delay, **(I)** heart malformations, and **(J)** neural tube closure defects in the spinal cord or in the cranial region. *N* values in top right of panel indicate total number of *Mtrr* ^*gt/gt*^ conceptuses assessed. Fisher’s exact test, **p < 0.01, ***p < 0.001. **(K**,**L)** Images of embryos (top panel) and placentas (bottom panel) from the same conceptus at E10.5 that belong to the phenotypic groups indicated in **(G**–**J)**. Phenotypically normal embryos and placentas from C57Bl/6J and *Mtrr* ^*gt/gt*^ conceptuses at E10.5 are also shown. The heart defect shown is pericardial edema (yellow arrowhead). The neural tube defect shown is an open neural tube in the cranial region (pink arrowhead) and both images show the same embryo in different orientations. Black arrowheads, allantois attachment site in placenta. Dotted line indicates approximate outline of labyrinth/chorion. Scale bars: **(A**–**C)** 500 μm, **(K**,**L)** 1 mm. Ch, chorion; DD, developmental delay; EPC, ectoplacental cone; FGR, fetal growth restriction; Ht, heart defect; MD, mesometrial decidua; NTD, neural tube defect; PN, phenotypically normal; P-TGCs, parietal trophoblast giant cells; SCM, severe conceptus misalignment; UL, uterine lumen remnant.

To better understand the eccentric placenta phenotype in greater detail, we examined centrally located transverse histological sections of C57Bl/6J and *Mtrr* ^*gt/gt*^ placentas at E8.5 after chorioallantoic attachment was complete (Cross et al., 2003). In all C57Bl/6J and *Mtrr* ^*gt/gt*^ placental sections assessed, the allantois was attached in a normal manner in the center of the chorion. However, a proportion of *Mtrr* ^*gt/gt*^ placentas displayed trophoblast compartments (including the chorion and EPC) that were noticeably askew within their decidual swellings (Figures 3A–C). The degree of skewed growth was determined by measuring the angle between the midline of the decidual swelling and the midline of the chorion or EPC (Figures 3A–C). In *Mtrr* ^*gt/gt*^ placentas, the angles of chorion and EPC skewing were proportionate with an average angle of 10.2° ± 10.5° (mean ± s.d.) and 8.3° ± 15.9°, respectively (Figures 3D–F). These values showed an increased trend compared to C57Bl/6J conceptuses (chorion: 2.4° ± 4.5° (Mann-Whitney test, *p* = 0.020); EPC: 0.3° ± 1.0°, *p* = 0.143; Figures 3D,E). Given the proportionality of chorion and EPC misalignment (Figure 3F), we hypothesized that the skewed orientation extended to the entire *Mtrr* ^*gt/gt*^ conceptus. Inter-individual variability of chorion/EPC skewing between *Mtrr* ^*gt/gt*^ conceptuses was apparent (Figures 3D,E) suggesting skewing occurred along a spectrum of severity. Therefore, we defined a chorion or EPC as “skewed” when its angle was >2 s.d. above the control mean (chorion: >11.4°; EPC: >2.4°; Figures 3D,E). Using this definition, 50.0% (6/12) of *Mtrr* ^*gt/gt*^ trophoblast compartments at E8.5 were skewed compared to only 14.3% (2/14) in C57Bl/6J conceptuses (Figures 3D,E). Given the spectrum of phenotypic severity, we propose that the eccentrically-located placenta phenotype identified during gross dissection likely denotes the extreme end of the skewed conceptus orientation spectrum. Moreover, less severe conceptus skewing was likely unappreciated during gross dissection.

### Embryonic Phenotypes Associate With Skewed Orientation in *Mtrr* ^*gt/gt*^ Conceptuses at E10.5

To determine whether embryonic phenotypes were associated with conceptus misalignment, we assessed our phenotyping data from *Mtrr* ^*gt/gt*^ conceptus dissections at E10.5 (Padmanabhan et al., 2013; Padmanabhan et al., 2017) for overlap between severe conceptus skewing (denoted as “eccentric placenta development” during gross dissection in this study and “eccentric chorioallantoic attachment” in a previous study (Padmanabhan et al., 2013)) and embryo growth defects or congenital malformations. Refer to our other published work for a full assessment of the individual phenotypic outcomes in *Mtrr* ^*gt/gt*^ conceptuses at E10.5 (Padmanabhan et al., 2013; Padmanabhan et al., 2017). We selected E10.5 for the time point of assessment due to the size of this data set (>215 *Mtrr* ^*gt/gt*^ conceptuses at E10.5) and because the detrimental effects of misaligned orientation on development might be more apparent at this stage. A caveat of this analysis was that only severe conceptus skewing was assessed using these criteria. While severe skewing did not associate with fetal growth restriction (5/15 misaligned *Mtrr* ^*gt/gt*^ embryos with 30–40 somite pairs had crown-rump lengths <2 s.d. of control mean, Fisher’s exact test, *p* = 0.193; Figures 3G,K,L), there was a correlation with developmental delay (9/18 misaligned *Mtrr* ^*gt/gt*^ embryos had <30 somite pairs, Fisher’s exact test, *p* = 0.003; Figures 3H,K,L). Separately, we observed that conceptuses with severe skewing showed an association with heart malformations including pericardial edema or reversed heart looping (9/18 of misaligned *Mtrr* ^*gt/gt*^ embryos had a heart abnormality; Fisher’s exact test, *p* < 0.0001; Figures 3I,K,L). Yet, none of the 18 *Mtrr* ^*gt/gt*^ embryos that failed to close their neural tube in either the cranial region (Figure 3L) or spinal cord region by E10.5 (Padmanabhan et al., 2013) displayed severe skewing (Figure 3J) indicating a lack of correlation between phenotypes. While a causal relationship is yet-to-be established, it is possible that skewed conceptus orientation might influence the rate of embryonic development and/or heart formation.

### Skewed Orientation Does Not Alter Trophoblast Differentiation of *Mtrr* ^*gt/gt*^ Conceptuses at E8.5

**FIGURE 4:**
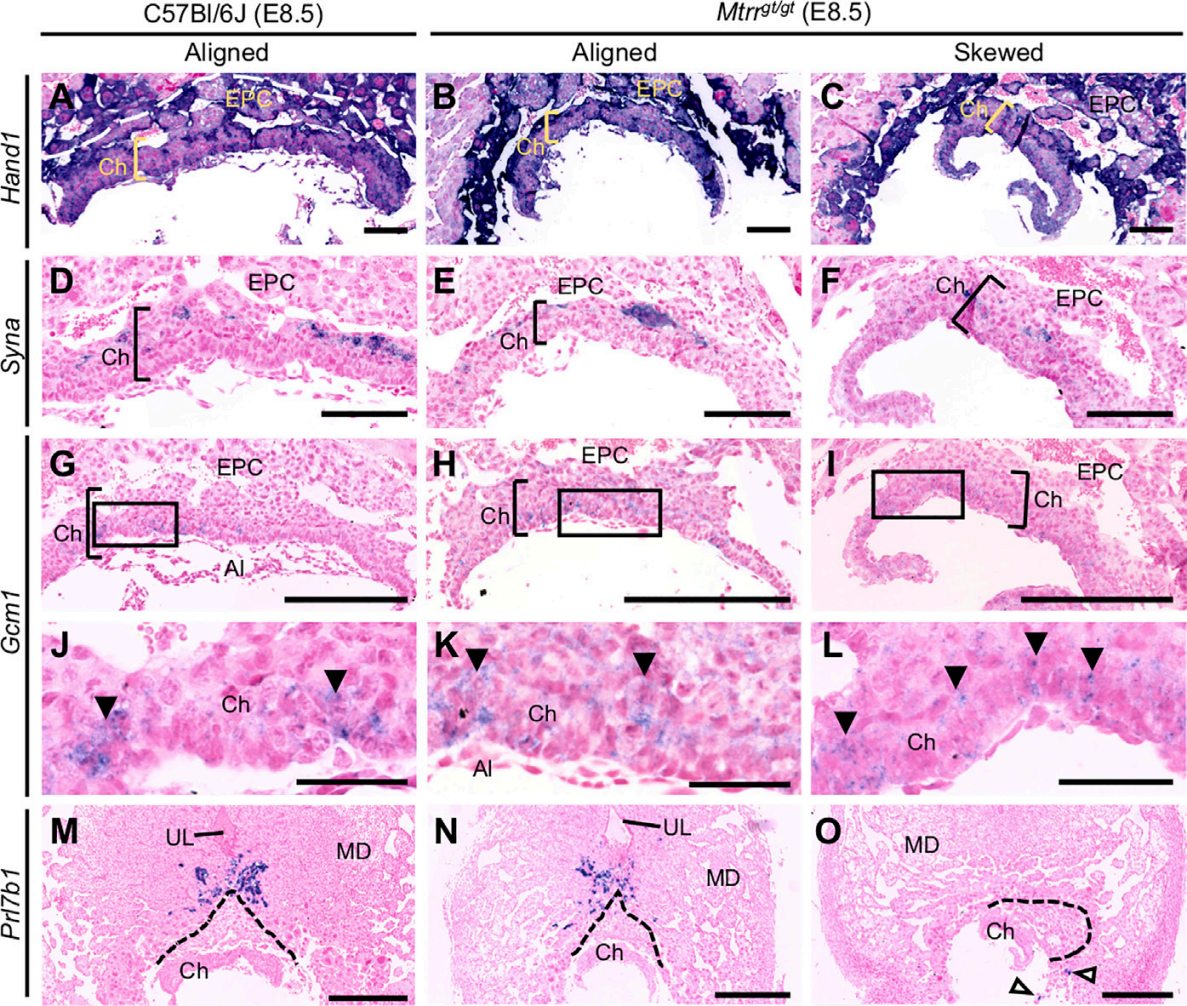
Early trophoblast differentiation occurs in *Mtrr* ^*gt/gt*^ mouse conceptuses at E8.5 regardless of misalignment. Analysis of trophoblast gene marker expression (purple) *via in situ* hybridization in C57Bl/6J (*N* = 6) and *Mtrr* ^*gt/gt*^ (*N* = 14) placentas at E8.5. *Mtrr* ^*gt/gt*^ placentas with aligned and skewed orientation are shown. Trophoblast markers included **(A**–**C)** *Hand1* (widespread trophoblast marker including sinusoidal TGC progenitors of the apical chorion), **(D**–**F)** *Syna* (indicates syncytiotrophoblast-I progenitors), **(G**–**L)** *Gcm1* (indicates syncytiotrophoblast-II progenitors; branch point initiation sites indicated by clusters of *Gcm1+* cells [black arrowheads]), and **(M**–**O)** *Prl7b1* (invasive trophoblast cells; white arrowheads indicate mislocated *Prl7b1* ^*+*^ cells). Dotted line indicates outer boundary of the ectoplacental cone. High magnification images in **(J**–**L)** represent boxed regions in **(G**–**I)**, respectively. Scale bars: **(A**–**C)** 1 mm, **(D**–**F)** 125 μm, **(G**–**I)** 250 μm, **(J**–**L)** 50 μm, **(M**–**O)** 500 μm. Al, allantois; Ch, chorion; EPC, ectoplacental cone; MD, mesometrial decidua; UL, uterine lumen remnant.

To explore whether skewed conceptus orientation altered the development of the trophoblast population, we performed *in situ* hybridization to detect the expression of key chorion and EPC trophoblast marker genes (Simmons et al., 2008). Histological sections of C57Bl/6J control (*N* = 6 placentas) and *Mtrr* ^*gt/gt*^ placentas (*N* = 14 placentas) were assessed at E8.5. *Mtrr* ^*gt/gt*^ placentas were subdivided into those with aligned and skewed orientation. Trophoblast marker gene expression of *Tpbpa* (EPC marker), *Hand1* (sinusoidal TGC progenitor and P-TGCs), *Syna* (syncytiotrophoblast-I progenitors) and *Gcm1* (syncytiotrophoblast-II progenitors) was present in all *Mtrr* ^*gt/gt*^ placentas assessed, regardless of the degree of skewing (Figures 3A–C, 4A–L). These data indicated that the trophoblast populations were present and patterned in *Mtrr* ^*gt/gt*^ placentas, and thus their formation was not greatly affected by skewed conceptus growth. However, *Prl7b1* mRNA expression (invasive trophoblast cell marker) was reduced in *Mtrr* ^*gt/gt*^ placentas with skewed orientation. The few *Prl7b1+* cells that were present were located in the lateral decidua rather than in the mesometrial decidua (Figures 4M–O). Mislocalization of invasive trophoblast cells might have significant implications for uterine spiral artery remodelling and placental function, and should be explored in future studies.

### *Mtrr* ^*gt/gt*^ Conceptus Misalignment Is Not Associated With Decidual Blood Sinus Area at E8.5

**FIGURE 5:**
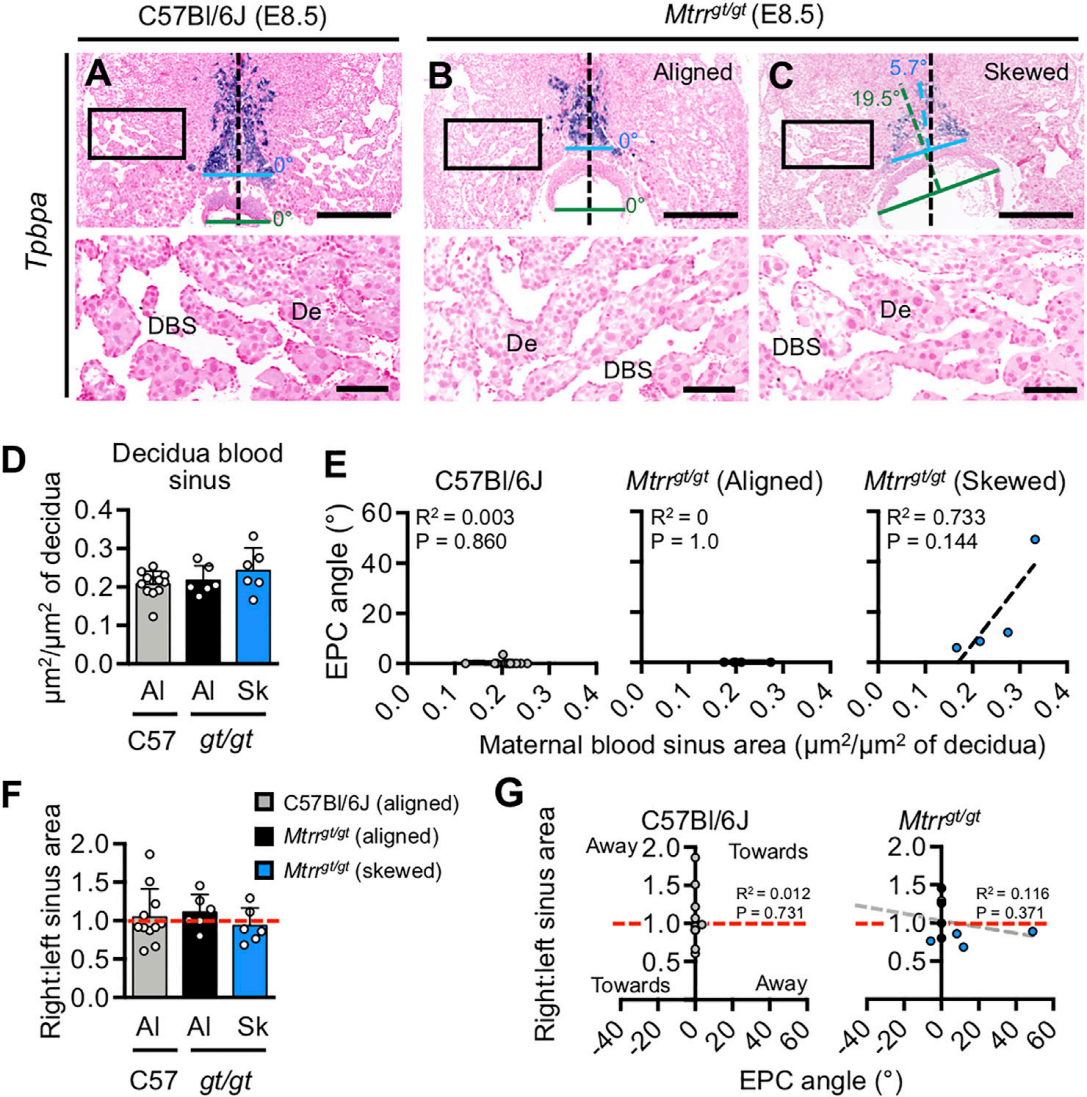
Mouse conceptus skewing does not correlate with decidual blood sinus area. **(A**–**C)** Histological sections of **(A)** C57Bl/6J and **(B**,**C)** *Mtrr* ^*gt/gt*^ placentas at E8.5. Ectoplacental cones were stained for *Tpbpa via in situ* hybridization. DNA, pink. **Bottom panel** represents higher magnification of boxed region in **top panel**. Placentas with **(A**,**B)** aligned and **(C)** skewed orientation are shown. *N* = 6–12 placentas/group were assessed. Black dashed line bisects the placenta. Blue dashed line bisects the EPC. Green dashed line bisects the chorion. Skewing angles are indicated for chorion (green) and EPC (blue). De, decidua; DBS, decidual blood sinus. Scale bars: **top panel**, 500 μm; **bottom panel**, 100 μm. **(D)** Graph showing average area of decidual blood sinuses (mean ± s.d.) per total decidua area assessed in histological sections of C57Bl/6J (grey bar) and *Mtrr* ^*gt/gt*^ (black and blue bars) placentas at E8.5. Aligned (Al) and skewed (Sk) placentas are represented (*N* = 6–12 placentas/group, with at least three sections assessed per placenta). One-way ANOVA, *p* = 0.2586. **(E)** Linear regression analyses of decidua blood sinus area in relation to EPC angle for C57Bl/6J and *Mtrr* ^*gt/gt*^ conceptuses. Aligned and skewed *Mtrr* ^*gt/gt*^ placentas were considered separately (*N* = 6–12 placentas/group). **(F)** Data depicting the ratio of decidua blood sinus area on the right and left sides of implantation sites from C57Bl/6J (C57) and *Mtrr* ^*gt/gt*^ (*gt/gt*) conceptuses at E8.5. Data represented as mean ± sd. Aligned (Al) and skewed (Sk) placentas were considered separately (*N* = 6–12 placentas/group). Dashed red line indicates a ratio of 1. One-way ANOVA, *p* = 0.6050. **(G)** Linear regression analysis to determine whether conceptus skewing occurred towards or away from the greatest decidual blood sinus area. The EPC angle and the ratio of right:left decidua blood sinus area were compared in C57Bl/6J and *Mtrr* ^*gt/gt*^ conceptuses at E8.5 (*N* = 6–12 conceptuses/group). Aligned (grey or black dots) and skewed (blue dots) conceptuses are shown. Dashed red line, right:left blood sinus area ratio of 1. Dashed grey line, line of best fit. *See* also Supplementary Figures 2A,B.

To better understand the cause of conceptus misalignment, we investigated a potential maternal effect of the *Mtrr* ^*gt*^ allele. Another model of skewed EPC growth exhibits dilated decidual blood sinuses (Winterhager et al., 2013). This association suggested that defects in the perfusion or structure of the maternal decidua might lead to skewed orientation of conceptus growth. Therefore, we measured the combined blood sinus area in the lateral and mesometrial decidual regions in centrally located transverse histological sections of C57Bl/6J and *Mtrr* ^*gt/gt*^ implantation sites at E8.5 (*N* = 6–12 placentas/group). Notably, the *Mtrr* ^*gt/gt*^ conceptuses were derived from an *Mtrr* ^*gt/gt*^ mother and father. Both aligned and skewed *Mtrr* ^*gt/gt*^ conceptuses were separately considered. The average proportion of the decidua attributed to blood sinuses was similar in C57Bl/6J, *Mtrr* ^*gt/gt*^ aligned, and *Mtrr* ^*gt/gt*^ skewed placentas (*p* = 0.259; Figure 4, Figures 5A–D). Furthermore, there was no correlation between decidual blood sinus area and chorion angle (*p* > 0.30; Supplementary Figure S2A) or EPC angle (*p* > 0.14; Figure 5E) in either *Mtrr* ^*gt/gt*^ group assessed compared to controls. Next, we examined whether left-right asymmetry in blood sinus area was evident and potentially guided the direction of conceptus misalignment. To do this, the ratio of right:left decidua blood sinus area was calculated. Like the controls, decidual blood sinus area was similar on the right and left sides of *Mtrr* ^*gt/gt*^ implantation sites, and was independent of whether conceptus skewing occurred (*p* = 0.605; Figure 5F). For each implantation site, the angle and direction of conceptus skewing was then compared to right-left decidual blood sinus ratio. Using histological sections of individual implantation sites, the chorion or EPC angle was classified as positive if it was skewed to the right and negative if it was skewed to the left. This value was plotted against the ratio of right:left decidual blood sinus area for that site (Figure 5G and Supplementary Figure S2B). In this model, a line-of-best-fit sloping downwards towards the right would indicate that the chorion/EPC angled away from the greater blood sinus area, and a slope upwards towards the right would indicate that the chorion/EPC angled towards the greater blood sinus area. In C57Bl/6J controls, chorion and EPC skewing was absent regardless of whether the blood sinus area was greater on the right or left (Figure 5G and Supplementary Figure S2B). Similarly in *Mtrr* ^*gt/gt*^ conceptuses, there was no statistically significant relationship between blood sinus size and the direction of chorion or EPC growth (*p* > 0.37; Figure 5G and Supplementary Figure S2B). These data suggested that the volume of maternal blood perfused into the implantation site was unlikely related to the orientation of *Mtrr* ^*gt/gt*^ conceptuses at E8.5.

### Conceptus Misalignment Is Associated With the *Mtrr* Genotype of the Maternal Grandparents

**FIGURE 6:**
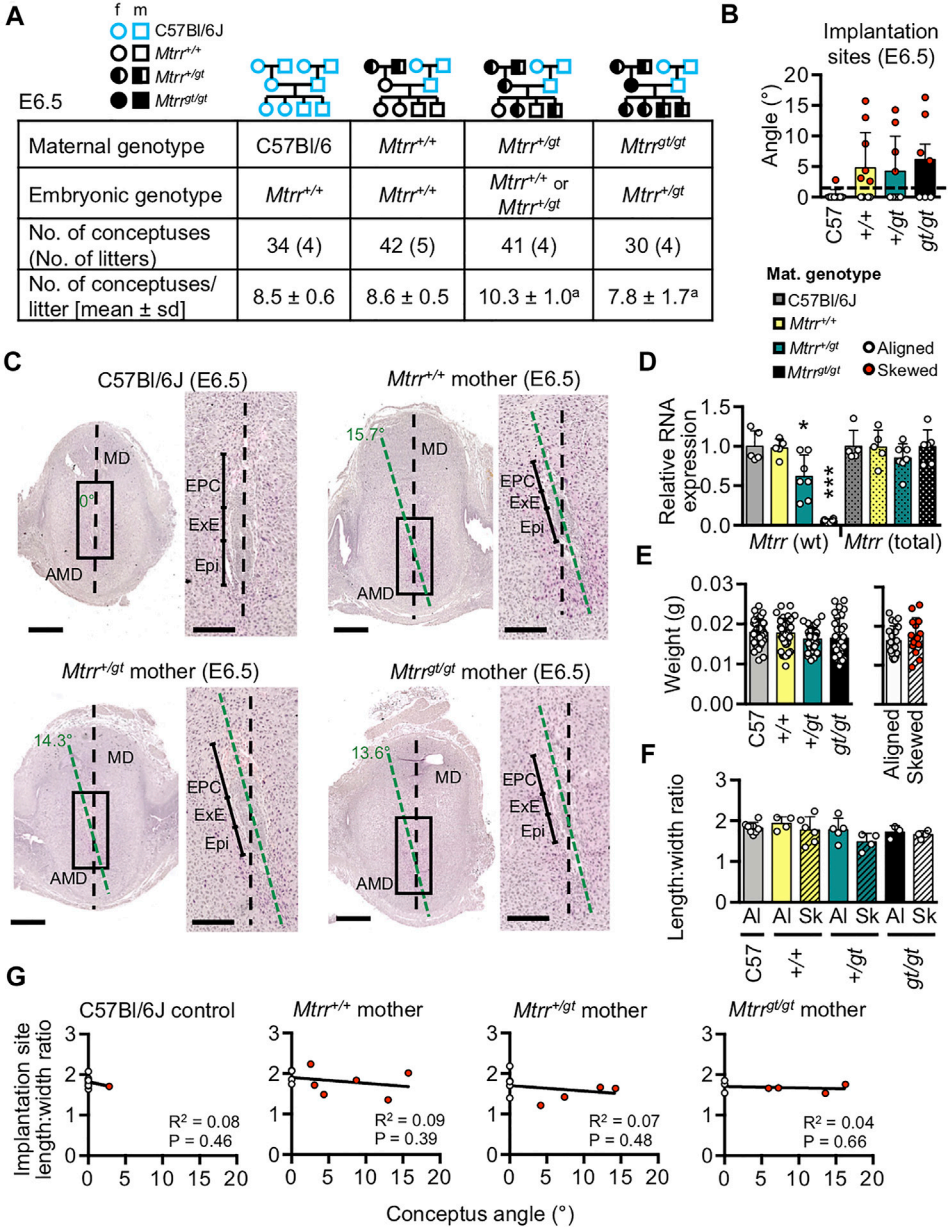
The effect of maternal *Mtrr* ^*gt*^ genotype on mouse conceptus skewing at E6.5. **(A)** Litter parameters at E6.5 obtained when manipulating the maternal *Mtrr* ^*gt*^ genotype. *N* = 4 litters/cross with 31–41 conceptuses/cross. Litter sizes: Ordinary one-way ANOVA, *p* = 0.0351, ^a^Dunn multiple comparison test: *Mtrr* ^*+/gt*^ versus *Mtrr* ^*gt/gt*^, *p* < 0.05. Pedigree key: circle, female; squares, males; blue outline, C57Bl/6J mouse strain; black outline, *Mtrr* ^*gt*^ mouse strain; white fill, *Mtrr* ^*+/+*^; half black/half white, *Mtrr* ^*+/gt*^; black fill, *Mtrr* ^*gt/gt*^. **(B)** Graph depicting the average angle of alignment at E6.5 in conceptuses derived from C57Bl/6J, *Mtrr* ^*+/+*^, *Mtrr* ^*+/gt*^, or *Mtrr* ^*gt/gt*^ mothers and C57Bl/6J fathers. White dots, aligned conceptuses. Red dots, conceptuses with angles >2 s.d. above the control mean (indicated by dashed black line). *N* = 7–10 conceptuses/group. Ordinary one-way ANOVA, *p* = 0.1438. **(C)** H&E stained histological sections of implantation sites at E6.5 derived from C57Bl/6J, *Mtrr* ^*+/+*^, *Mtrr* ^*+/gt*^, or *Mtrr* ^*gt/gt*^ mothers and C57Bl/6J fathers (*N* = 7–10 conceptuses/group). Black dashed line bisects the implantation site. Green dashed line bisects conceptus. Angles indicate the degree of conceptus skewing. **Right-hand panel** indicates higher magnification of boxed region in **left-hand panel**. Scale bars, low magnification, 500 μm; high magnification, 250 μm. AMD, antimesometrial decidua; EPC, ectoplacental cone; Epi, epiblast; ExE, extra-embryonic ectoderm; MD, mesometrial decidua. **(D)** RT-qPCR analysis of relative RNA levels (mean ± s.d.) of the wildtype (wt) *Mtrr* transcripts and total *Mtrr* transcripts (wt + gene-trapped transcripts) in whole implantation sites at E6.5 derived from C57Bl/6J, *Mtrr* ^*+/+*^, *Mtrr* ^*+/gt*^ and *Mtrr* ^*gt/gt*^ mothers and C57Bl/6J fathers. *N* = 5–7 implantation sites/group. Values were relative to C57Bl/6J (normalized to 1). One-way ANOVA with Tukey’s multiple comparison test, *p < 0.05, ***p < 0.001. **(E)** **Left-hand graph:** average weights of whole implantation sites at E6.5 (mean ± s.d.) derived from C57Bl/6J, *Mtrr* ^*+/+*^, *Mtrr* ^*+/gt*^, or *Mtrr* ^*gt/gt*^ mothers and C57Bl/6J fathers. *N* = 30–42 conceptuses/group. Alignment not determined. Maternal genotypes are indicated. One-way ANOVA, *p* = 0.114. **Right-hand graph:** conceptus weights from *Mtrr* ^*+/+*^, *Mtrr* ^*+/gt*^, and *Mtrr* ^*gt/gt*^ mothers were pooled and divided into aligned orientation (white dots, *N* = 20 conceptuses) and skewed orientation (red dots; *N* = 14 conceptuses). Independent *t* test, *p* = 0.392. **(F)** Implantation site shape as determined by the ratio of decidua length (along the mesometrial-antimesometrial axis) and width (along the lateral axis). Maternal genotypes are indicated. Aligned (Al) and skewed (Sk) conceptuses were considered separately (*N* = 3–8 implantation sites/group). Data presented as mean ± sd. One-way ANOVA, *p* = 0.1371. **(G)** Linear regression analysis between conceptus angle and decidua length:width ratio for conceptuses at E6.5 derived from C57Bl/6J, *Mtrr* ^*+/+*^, *Mtrr* ^*+/gt*^, and *Mtrr* ^*gt/gt*^ mothers (*N* = 7–10 conceptuses/group). White dots, aligned conceptuses; red dots, skewed conceptuses; black line, line of best fit.

To more directly consider a maternal effect on conceptus alignment, we manipulated the maternal *Mtrr* genotype in highly controlled genetic pedigrees and assessed the frequency of conceptus skewing. To do this, *Mtrr* ^*+/+*^, *Mtrr* ^*+/gt*^, and *Mtrr* ^*gt/gt*^ females were mated to C57Bl/6J control males. Implantation sites were dissected at E6.5 (*N* = 31–41 conceptuses/maternal genotype from four litters) and assessed for conceptus skewing and defects in decidualization (*see* below). C57Bl/6J intercrosses were used as controls. The average litter size was significantly different between the pedigrees (Figure 6A; One-way ANOVA, *p* = 0.0351) with greatest difference between litters from *Mtrr* ^*+/gt*^ females (10.3 ± 1.0 conceptuses/litter [mean ± s.d.]) and *Mtrr* ^*gt/gt*^ females (7.8 ± 1.7 conceptuses/litter, Dunn’s multiple comparison, *p* < 0.05). To assess the extent of conceptus skewing caused by the maternal *Mtrr* ^*gt*^ genotype at E6.5, centrally located transverse histological sections of implantation sites at E6.5 were examined (*N* = 7–10 implantation sites/group). In a manner similar to the analysis at E8.5 (Figures 3A–C), the angle between the midlines of the implantation site and the entire conceptus was measured (Figures 6B,C). Most C57Bl/6J conceptuses were aligned along the mesometrial-antimesometrial axis with an average angle of 0.3° ± 0.9° (mean ± s.d.; Figures 6B,C). In contrast, conceptuses derived from *Mtrr* ^*+/+*^, *Mtrr* ^*+/gt*^ and *Mtrr* ^*gt/gt*^ females showed average angles that were greater than controls (*Mtrr* ^*+/+*^ mothers: 4.7° ± 5.8°; *Mtrr* ^*+/gt*^ mothers: 4.2° ± 5.7°; *Mtrr* ^*gt/gt*^ mothers: 6.2° ± 6.7°; Figure 6B). Statistical significance was not reached due to high inter-individual variability (Figure 6B). However, similar to our observations in *Mtrr* ^*gt/gt*^ conceptuses at E8.5 (Figures 3D,E), skewing at E6.5 (regardless of maternal *Mtrr* genotype) occurred across a spectrum of severity (Figure 6B). Therefore, we similarly defined a conceptus at E6.5 as “skewed” when it had an angle >2 s.d. above the control mean (i.e., >2.2°). Using this definition, only 11% (1/9) of C57Bl/6J conceptuses were skewed compared to 60% (6/10), 44% (4/9), and 57% (4/7) of conceptuses with *Mtrr* ^*+/+*^, *Mtrr* ^*+/gt*^, and *Mtrr* ^*gt/gt*^ mothers, respectively (Figure 6B). Altogether, these data provided evidence that skewed conceptus growth was established earlier than E6.5, potentially at implantation. Furthermore, there was a similar frequency of misaligned conceptuses derived from *Mtrr* ^*+/+*^ mothers as from *Mtrr* ^*+/gt*^ and *Mtrr* ^*gt/gt*^ mothers, implicating the *Mtrr* ^*+/gt*^ maternal grandparents in the inheritance of this phenotype. Multigenerational inheritance of developmental phenotypes was previously reported in the *Mtrr* ^*gt*^ model in other contexts (Padmanabhan et al., 2013; Padmanabhan et al., 2017; Padmanabhan et al., 2018) and will be explored further below.

### Gross Decidual Morphology Is Unchanged by Maternal *Mtrr* ^*gt*^ Allele

Other studies showed that skewed conceptus orientation might result from abnormal decidual remodeling and signaling (Alexander et al., 1996; Zhang et al., 2014). Therefore, we assessed the effects of the maternal *Mtrr* ^*gt*^ mutation on decidualization. First, to understand the degree of *Mtrr* knockdown caused by the *Mtrr* ^*gt*^ allele in the decidua, wildtype *Mtrr* transcript levels were determined *via* RT-qPCR in whole implantation sites at E6.5 as a proxy for decidual cells (*N* = 5–7 whole implantation sites/group). Implantation sites at E6.5 consist largely of decidualized stroma with a relatively low contribution by fetal-derived cells. This experiment was important because the extent of wildtype *Mtrr* gene knockdown in *Mtrr* ^*+/gt*^ and *Mtrr* ^*gt/gt*^ cells is tissue specific (Elmore et al., 2007; Padmanabhan et al., 2013; Sowton et al., 2020). As expected, implantation sites from *Mtrr* ^*+/+*^ mothers displayed a similar level of wildtype *Mtrr* transcripts as C57Bl/6J controls (98.0% of control levels; Figure 6D). Furthermore, this level was reduced to 62.0 and 6.0% of controls in implantation sites from *Mtrr* ^*+/gt*^ and *Mtrr* ^*gt/gt*^ mothers, respectively (*p* < 0.0001; Figure 6D), and was within the range observed in other *Mtrr* ^*+/gt*^ and *Mtrr* ^*gt/gt*^ tissues (Elmore et al., 2007; Padmanabhan et al., 2013; Sowton et al., 2020). Total *Mtrr* transcript levels (includes *Mtrr* ^+^ and *Mtrr* ^*gt*^ RNA) were similar across all maternal genotypes (*p* = 0.4941; Figure 6D) indicating that transcriptional compensation for wildtype *Mtrr* deficiency did not occur.

Next, gross decidual morphology was assessed in histological sections of implantation sites at E6.5. The extent of decidualization was unaffected by a maternal *Mtrr* ^*gt*^ allele at E6.5 since the average implantation site weight did not differ between control and *Mtrr* ^*+/+*^, *Mtrr* ^*+/gt*^, or *Mtrr* ^*gt/gt*^ mothers (Figure 6E; *p* = 0.114). Furthermore, implantation site weight was similar between aligned and skewed conceptuses regardless of genotype (Figure 6E; *p* = 0.393), and there was no apparent correlation between implantation site weight and degree of conceptus skewing (Supplementary Figures S1A,B). No litter size effect on implantation site weight at E6.5 was observed (Supplementary Figure S1C). Typically, an implantation site is ellipsoid with the mesometrial-antimesometrial axis (length) showing a longer dimension than the lateral axis (width). A rounder implantation site might indicate defective decidualization (Alexander et al., 1996). Centrally located transverse sections of implantation sites from C57Bl/6J mothers and from *Mtrr* ^*+/+*^, *Mtrr* ^*+/gt*^, or *Mtrr* ^*gt/gt*^ mothers with aligned or skewed conceptuses (*N* = 3–8 implantation sites/group) were measured along both axes. No significant differences in decidua length:width ratios were observed in implantation sites from any maternal genotypic group compared to controls regardless of skewing (*p* = 0.137; Figure 6F). Furthermore, there was no association between degree of conceptus skewing and implantation site dimensions (*p* > 0.39; Figure 6G). When lateral decidual cell density was quantified by nuclear counts in implantation sites containing skewed conceptuses derived from *Mtrr* ^*+/+*^, *Mtrr* ^*+/gt*^, or *Mtrr* ^*gt/gt*^ mothers (*N* = 4–9 implantation sites/group), there was no significant difference compared to C57Bl/6J conceptuses (*p* = 0.170; Supplementary Figures 2C,D). Altogether, gross decidual morphology was unaffected by a maternal *Mtrr* ^*gt*^ allele.

### Signaling Pathways in Decidua Are Potentially Disrupted by the *Mtrr* ^*gt*^ Mutation

**FIGURE 7:**
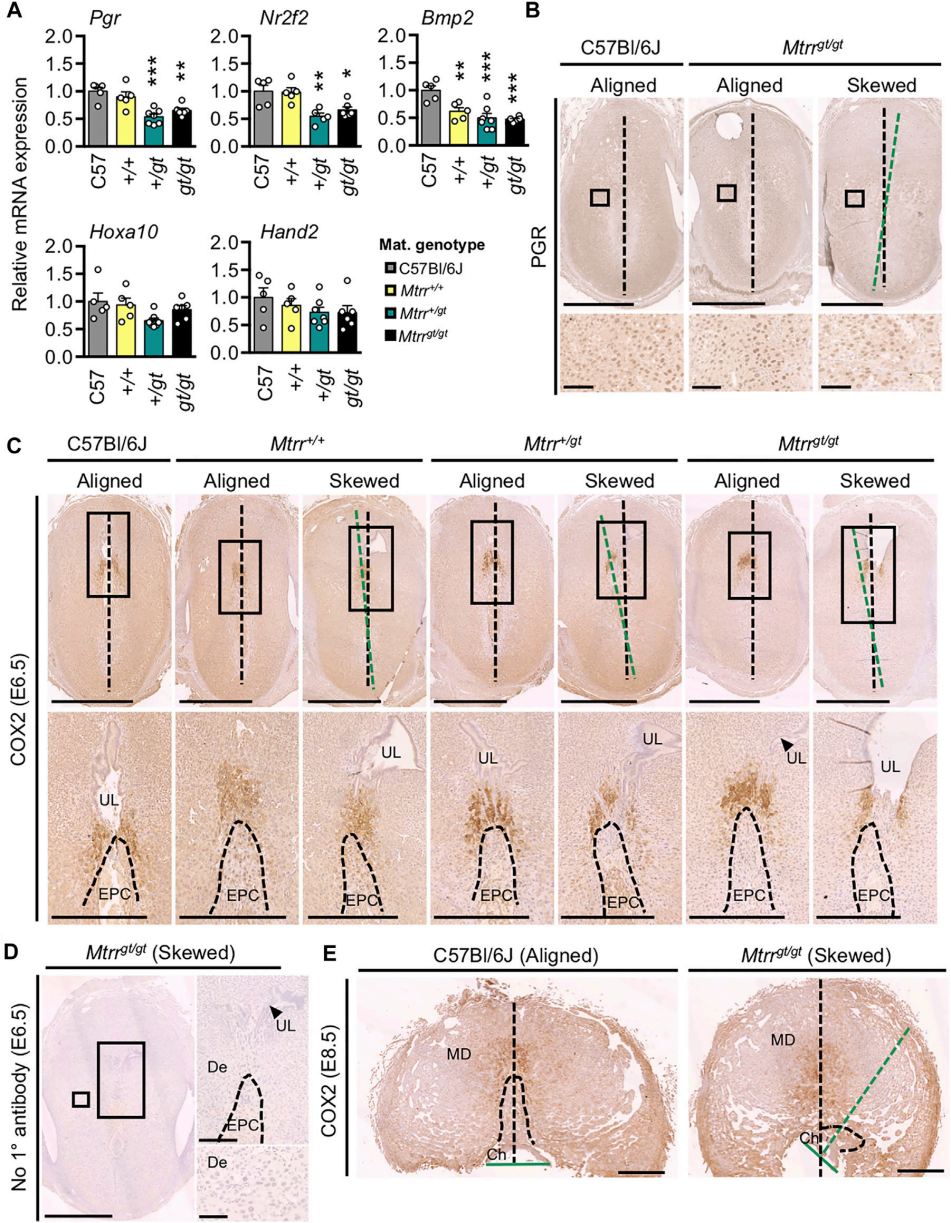
Maternal and grandparental *Mtrr* ^*gt*^ allele might affect molecular signaling in mouse decidua at E6.5. **(A)** RT-qPCR analysis of decidualization gene markers in whole implantation sites at E6.5 derived from C57Bl/6J, *Mtrr* ^*+/+*^, *Mtrr* ^*+/gt*^ or *Mtrr* ^*gt/gt*^ mothers (*N* = 5–7 sites/group). Data presented as mean ± s.d., relative to C57Bl/6J controls (normalized to 1). One-way ANOVA with Tukey’s multiple comparison test, *p < 0.05, **p < 0.01, ***p < 0.001. **(B)** Progesterone receptor (PGR) immunostaining in implantation sites at E6.5 derived from C57Bl/6J mothers and aligned and skewed conceptuses from *Mtrr* ^*gt/gt*^ mothers. *N* = 3 sites/genotype were assessed. **(C)** COX2 immunostaining (brown) in implantation sites at E6.5 derived from C57Bl/6J, *Mtrr* ^*+/+*^, *Mtrr* ^*+/gt*^, or *Mtrr* ^*gt/gt*^ mothers and C57Bl/6J fathers. N = 2–5 sites/maternal genotype were assessed. DNA, blue. Both aligned and skewed conceptuses are shown. In panels **(B**,**C)**, the degree of skewing is indicated by green dotted line that bisects the conceptus compared to black dotted line that bisects the decidual swelling. **(D)** No primary antibody control in a skewed *Mtrr* ^*gt/gt*^ implantation site at E6.5. DNA, blue. **(E)** COX2 immunostaining (brown) in an aligned C57Bl/6J placenta and skewed *Mtrr* ^*gt/gt*^ placenta at E8.5. *N* = 6 sites/genotype. DNA, blue. **(B**–**E)** Boxed area indicates region of higher magnification in adjacent panel. Black dotted line bisects the implantation site. Green dotted line bisects the conceptus (E6.5) or chorion (E8.5). Maternal genotype is indicated. Scale bars: **(B)** 500 μm, **(C)** top, 1 mm; bottom, 500 μm, **(D)** left, 1 mm; top right, 500 μm; bottom right, 100 μm, **(E)** 1 mm. Ch, chorion; De, decidua; EPC, ectoplacental cone (dashed line indicates boundary); MD, mesometrial decidua; UL, uterine lumen remnant.

Next, we explored the effects of maternal *Mtrr* ^*gt*^ allele on the molecular regulation of decidualization. Molecular markers of decidualization were assessed by RT-qPCR in whole implantation sites at E6.5 from C57Bl/6J, *Mtrr* ^*+/+*^, *Mtrr* ^*+/gt*^, and *Mtrr* ^*gt/gt*^ mothers (*N* = 5–7 sites/group). Progesterone receptor (PGR) (Lydon et al., 1995) and its downstream effectors NR2F2 (Wetendorf and DeMayo, 2012) and BMP2 (Lee et al., 2007) are indispensable for decidualization. While *Pgr* and *Nr2f2* mRNA expression was significantly reduced in the implantation sites from *Mtrr* ^*+/gt*^ and *Mtrr* ^*gt/gt*^ mothers compared to C57Bl/6J and *Mtrr* ^*+/+*^ mothers (*p* < 0.0012; Figure 7A), transcript levels of downstream targets of PGR signalling including *Hand2* and *Hoxa10* (Lim et al., 1999; Okada et al., 2014) were within the normal range (*p* = 0.4132 and *p* = 0.0796, respectively; Figure 7A). The degree of conceptus skewing was unknown in the samples assessed since each implantation site was snap frozen immediately after dissection, whereas analysis of conceptus alignment at this stage was determined in histological sections. However, immunohistochemistry suggested subtly lower PGR protein levels in both *Mtrr* ^*gt/gt*^ aligned and skewed conceptuses compared to C57Bl/6J controls (Figure 7B) though this decrease in protein expression might be insufficient to alter downstream effectors. Future studies should include protein quantification of PGR and downstream targets. Notably, *Bmp2* transcripts were significantly decreased in implantation sites from all three *Mtrr* maternal genotypes relative to controls (*p* = 0.0001; Figure 7A). Low *Bmp2* transcript levels in *Mtrr* ^*+/+*^ conceptuses derived from *Mtrr* ^*+/+*^ mothers that associated with normal *Pgr* transcripts (Figure 7A) and normal decidualization (Supplementary Figures 2C,D) suggested that potential BMP2 signalling defects might occur independent of progesterone signalling and decidualization defects. Further experiments are required to elucidate the specific role of BMP2 signalling in the establishment of conceptus orientation.

### Decidual COX2 Expression Is Normal in Implantation Sites With Skewed Orientation

When the gene encoding for COX2 protein is knocked out, defects in implantation and decidualization result (Lim et al., 1999). COX2 is important for prostaglandin synthesis and is initially expressed in the decidua adjacent to the implanting embryo at E4.5 followed by restricted expression in the mesometrial decidua by E5.5 and at E7.5 (Chakraborty et al., 1996). Aberrant COX2 expression was also observed in other models of conceptus misorientation (Daikoku et al., 2011; Cha et al., 2014; Zhang et al., 2014). Therefore, we investigated COX2 protein expression in the decidua at E6.5 from *Mtrr* ^*+/+*^, *Mtrr* ^*+/gt*^, and *Mtrr* ^*gt/gt*^ mothers (mated to C57Bl/6J males), and at E8.5 from *Mtrr* ^*gt/gt*^ mothers (mated to *Mtrr* ^*gt/gt*^ males), particularly in association with aligned and skewed conceptus orientation. Within centrally located transverse histological sections, COX2 immunostaining in C57Bl/6J controls was restricted to the mesometrial decidua above the tip of the EPC, in line with the antimesometrial-mesometrial axis (Figures 7C,E). A similar pattern was observed in implantation sites from *Mtrr* ^*+/+*^, *Mtrr* ^*+/gt*^ and *Mtrr* ^*gt/gt*^ mothers, regardless of the extent of conceptus skewing (Figure 7C). Strikingly, the most skewed *Mtrr* ^*gt/gt*^ conceptus at E8.5 showed normal mesometrial decidual patterning of COX2 even though the EPC was more laterally located (Figure 7E). These data suggested that general decidual patterning was established and that COX2 expression did not dictate conceptus orientation in the *Mtrr* ^*gt*^ model.

### Conceptus Skewing Is Transgenerationally Inherited in the *Mtrr* ^*gt*^ Mouse Line

**FIGURE 8:**
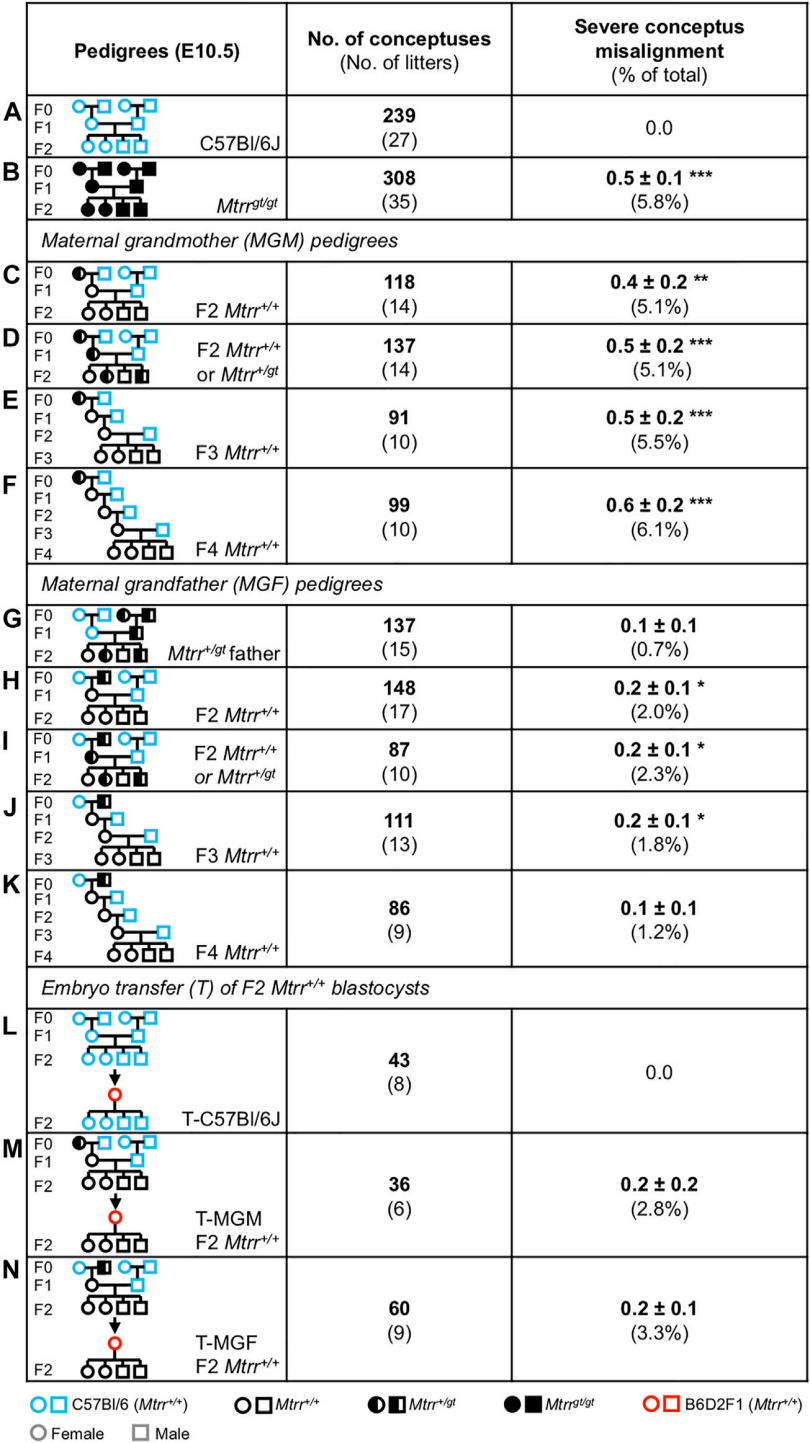
Severe conceptus misalignment is transgenerationally inherited in the *Mtrr* ^*gt*^ mouse line *via* the maternal grandparental lineage. **(A**–**K)** Frequency of severe conceptus misalignment at E10.5 caused by the **(C**–**F)** *Mtrr* ^*+/gt*^ maternal grandmother (MGM) or **(H**–**K)** *Mtrr* ^*+/gt*^ maternal grandfather (MGF) compared to **(A)** C57Bl/6J conceptuses. **(B)** *Mtrr* ^*gt/gt*^ and **(G)** *Mtrr* ^*+/gt*^ paternal pedigrees were also assessed. **(L**–**N)** Frequency of severe conceptus misalignment at E10.5 after the transfer of wildtype pre-implantation embryos derived from **(M)** an *Mtrr* ^*+/gt*^ MGM and *Mtrr* ^*+/+*^ mother (T-MGM) or **(N)** an *Mtrr* ^*+/gt*^ MGF and *Mtrr* ^*+/+*^ mother (T-MGF) into a B6D2F1 pseudopregnant recipient female. **(L)** C57Bl/6J embryos were transferred as a control. This experiment was originally performed in (Padmanabhan et al., 2013). Data is displayed as the average number of phenotypically affected conceptuses/litter (±s.e.m.) followed by the percentage of total conceptuses assessed in brackets. Independent *t* test compared to respective C57Bl/6J controls, *p < 0.05, **p < 0.01, ***p < 0.001. Pedigree key: circle, female; squares, males; blue outline, C57Bl/6J mouse strain; red outline, B6D2F1 mouse strain; black outline, *Mtrr* ^*gt*^ mouse strain; white fill, *Mtrr* ^*+/+*^; black fill, *Mtrr* ^*gt/gt*^; half black/half white, *Mtrr* ^*+/gt*^.

Since conceptus skewing was apparent from E6.5 in *Mtrr* ^*+/+*^ conceptuses derived from *Mtrr* ^*+/+*^ parents and *Mtrr* ^*+/gt*^ grandparents (Figures 6B,C, 7A), we explored a multigenerational effect on conceptus orientation. The *Mtrr* ^*gt*^ mouse line is known for its multigenerational effects on development specifically through the maternal grandparental lineage (Padmanabhan et al., 2013; Blake et al., 2021). To this end, we reanalyzed our published phenotype data set at E10.5 whereby several genetic pedigrees were established to rigorously assess the phenotypic effects of a maternal grandparental *Mtrr* ^*gt*^ allele on the subsequent wildtype generations (Padmanabhan et al., 2013; Padmanabhan et al., 2017). Additional litters were included where possible. The frequency of the phenotype previously referred to as “eccentric chorioallantoic attachment” (Padmanabhan et al., 2013) and re-characterised in this study as severe conceptus skewing was used as an indicator of conceptus misalignment. While assessing severe misalignment alone does not give a full picture of the extent of skewing in these pedigrees, the number of conceptuses assessed (*N* = 86–308 conceptuses/pedigree) allowed for statistical analysis. The breeding scheme was as follows (Figure 8): An F0 *Mtrr* ^*+/gt*^ female or male mouse was crossed with a control C57Bl/6J mate. Their wildtype adult daughters (i.e., the F1 generation) were selected for mating with a C57Bl/6J male and the wildtype grandprogeny (i.e., the F2 generation) were dissected and assessed at E10.5. Continuing a similar mating scheme allowed us to also assess the wildtype F3 and F4 generations. C57Bl/6J crosses were analyzed as a control.

Overall, a low frequency of severe conceptus skewing was observed in the F2 *Mtrr* ^*+/+*^ progeny of the *Mtrr* ^*+/gt*^ maternal grandmother (MGM) pedigree (5.1% of conceptuses, *p* < 0.01; Figure 8C) and the *Mtrr* ^*+/gt*^ maternal grandfather (MGF) pedigree (2.0% of conceptuses, *p* < 0.05; Figure 8H). This phenotype was not observed in the controls (Figure 8A). Notably, when the *Mtrr* genotype of the F1 female was altered (e.g., F1 *Mtrr* ^*+/+*^ or F1 *Mtrr* ^*+/gt*^ females), there was no impact on the frequency of severe skewing in the F2 *Mtrr* ^*+/+*^ progeny in either pedigree (MGM: Figure 8C versus Figure 8D; MGF: Figure 8H versus Figure 8I). This result indicated that the *Mtrr* ^*gt*^ genotype in the maternal grandparent was most important for conceptus misalignment, an observation consistent with other phenotypes (Padmanabhan et al., 2013).

There was a negligible paternal effect of the *Mtrr* ^*gt*^ allele on conceptus misalignment since this phenotype was rare in the direct offspring of an *Mtrr* ^*+/gt*^ male (0.7% of conceptuses, *p* = 0.183 compared to controls; Figures 8A,G). Since *Mtrr* ^*+/gt*^ males were derived from *Mtrr* ^*+/gt*^ parents (Figure 8G), this result also emphasized that a paternal grandparental effect was unlikely. Importantly, an *Mtrr* ^*gt*^ allele in a male was sufficient to cause conceptus misalignment in the F2 *Mtrr* ^*+/+*^ grandprogeny *via* his F1 *Mtrr* ^*+/+*^ daughters (Figure 8H), and highlighted the potential importance of the F1 uterine environment (*see* below). Since *Mtrr* ^*gt/gt*^ conceptuses at E10.5 derived from *Mtrr* ^*gt/gt*^ intercrosses showed a similar frequency of skewing (5.8% of conceptuses, *p* < 0.001) as F2 *Mtrr* ^*+/+*^ conceptuses from the MGM pedigree (Figure 8B versus Figure 8C), the mechanism was likely due to *Mtrr* deficiency in one (or both) maternal grandparent. Notably, the frequency of skewing was greater in F2 *Mtrr* ^*+/+*^ progeny when derived from an *Mtrr* ^*+/gt*^ MGM compared to an *Mtrr* ^*+/gt*^ MGF (Figure 8C versus Figure 8H) suggesting that the causative mechanism might be different when initiated *via* the F0 oocyte versus the F0 sperm, as was previously hypothesized (Blake et al., 2021). In further support of this hypothesis, we observed that severe conceptus misalignment occurred until at least until the F4 wildtype generation when derived from an F0 *Mtrr* ^*+/gt*^ female (Figures 8E,F) yet only until the F3 wildtype generation when derived from an F0 *Mtrr* ^*+/gt*^ male (Figures 8J,K). Phenotypic inheritance to the F3 generation and beyond indicates transgenerational epigenetic inheritance as a mechanism for conceptus misalignment in both pedigrees (Padmanabhan et al., 2013; Blake and Watson, 2016). Of note, the resolution of a transgenerationally-inherited phenotype (e.g., conceptus misalignment in the MGF pedigree) has not been previously reported in the *Mtrr* ^*gt*^ model. Overall, the effects of abnormal folate metabolism caused by the *Mtrr* ^*gt*^ mutation persisted for several wildtype generations with detrimental effects on conceptus alignment.

### Severe Conceptus Misalignment in the *Mtrr* ^*gt*^ Model Might Be Independent of the Uterus

Lastly, to determine whether the uterine environment influenced conceptus alignment, a published embryo transfer experiment was reanalyzed (Padmanabhan et al., 2013). In this case, pre-implantation F2 *Mtrr* ^*+/+*^ embryos were collected at E3.25 from the oviducts and uteri of F1 *Mtrr* ^*+/+*^ females (MGM or MGF pedigree) and transferred into the normal uteri of B6D2F1 pseudopregnant females (Figures 8M,N). C57Bl/6J embryos were transferred as a control (Figure 8L). The transferred embryos were allowed to implant and conceptuses were dissected at E10.5 for phenotypic analysis (Padmanabhan et al., 2013). Our hypothesis was if severe conceptus misalignment was absent at E10.5 after embryo transfer, then an abnormal uterine environment in the original F1 *Mtrr* ^*+/+*^ donor female was the cause of this phenotype. However, if severe misalignment was observed in the transferred conceptuses, then the defect occurred independent of the peri- or post-implantation uterine environment. We reported that some of the developmental phenotypes observed in F2 *Mtrr* ^*+/+*^ conceptuses persisted after embryo transfer including congenital malformations (mechanism independent of F1 uterine environment) but not fetal growth restriction or developmental delay, which were rescued (mechanism dependent on F1 uterine environment) (Padmanabhan et al., 2013).

Here, our analysis revealed that severe conceptus misalignment persisted after embryo transfer suggesting that this phenotype occurred independent of the F1 *Mtrr* ^*+/+*^ uterine environment (Figures 8L–N) (Padmanabhan et al., 2013). The percentage of transferred F2 *Mtrr* ^*+/+*^ conceptuses that were affected was similar to the non-transferred F2 *Mtrr* ^*+/+*^ conceptuses (Figures 8M,N versus Figures 8C,H). While the phenotypic frequency did not reach statistical significance, this was likely due to the low number of transferred embryos analyzed (*N* = 36–60 embryos from six to nine litters; Figures 8M,N). None of the transferred C57Bl/6J control conceptuses exhibited severe skewing (Figure 8L) indicating that the embryo transfer protocol itself was not responsible for this phenotype. Overall, we propose that conceptus misalignment in the *Mtrr* ^*gt*^ model occurred independent of a peri- and/or post-implantation uterine environment effect. Alternatively, a defect intrinsic to the embryo is likely.

## Discussion

We have shown here that abnormal folate metabolism due to the *Mtrr* ^*gt*^ mutation in mice influences conceptus orientation and spacing within the uterus. These phenotypes have implications for subsequent embryo and placenta development. Our data highlight the complexity of phenotype establishment associated with abnormal folate metabolism, with the mechanism acting beyond a direct maternal effect. We propose that conceptus misalignment and twinning are caused by an embryo-specific defect rather than a defect in the peri-/post-implantation uterine environment. We also implicate the *Mtrr* ^*gt*^ genotype of either maternal grandparent as the initial effector of conceptus misalignment, which persists at least until the wildtype great grandprogeny generation and exemplifies a transgenerationally inherited phenotype. In general, the mechanism of transgenerational epigenetic inheritance is not well understood and will take additional studies to elucidate.

Given the evidence presented here, conceptus misalignment can negatively impact embryo and placenta development. The degree of skewing occurred over a continuum, though whether minor skewing is harmful to development is currently unclear as we were only able to assess the consequences of severe skewing. Even though normal trophoblast lineage patterning (Simmons et al., 2008) occurred in severely skewed *Mtrr* ^*gt/gt*^ conceptuses at E8.5, an improperly oriented placenta would likely have insufficient access to maternal blood for normal feto-placental development as supported by the mislocalisation of spiral artery-remodeling *Prl7b1* ^+^ trophoblast cells. Severe conceptus skewing at E10.5 was also associated with embryonic developmental delay and heart malformations. Others have proposed the placenta-heart axis whereby that primary placental phenotype can result in secondary cardiovascular abnormalities (Barak et al., 1999; Hemberger and Cross, 2001; Perez-Garcia et al., 2018), and vice versa (Outhwaite et al., 2019). Whether this phenomenon occurs in skewed conceptuses is unclear and requires more careful consideration of heart development in the *Mtrr* ^*gt*^ model from earlier stages and investigation by conditional mutation or tetraploid aggregation experiments that isolate the consequences of heart-only or placenta-only knockdown of *Mtrr*. Notably, neural tube defects, which are associated with maternal folate deficiency in humans (MRC Vitamin Study Research Group, 1991) and occur in the *Mtrr* ^*gt/gt*^ embryos at E10.5 with incomplete penetrance (Padmanabhan et al., 2013), did not correlate with conceptus misalignment in our study suggesting a different causative mechanism.

Dizygotic twinning is a rare event in mice (De Clercq et al., 2019) with few reports of this phenomenon in the literature. While the cause is currently unknown, we hypothesize that dizygotic twinning is independent of the number of ovulations and results from poor blastocyst spacing prior to implantation. Thereafter, twinned embryos with overlapping development share one decidual swelling and display placental fusion as gestation progresses, similar to observations in other studies (De Clercq et al., 2019; Lopez-Tello and Sferruzzi-Perri, 2021). Blastocyst spacing in the mouse uterus is not well understood, yet it is a highly regulated event requiring mechanical processes in the uterus (e.g., muscular contractions and ciliary movement) and molecular signaling (e.g., LIF, ephrin A) between the uterus and blastocyst (Chen et al., 2013; Shi et al., 2014; Flores et al., 2020; Fujiwara et al., 2020). Manipulation of one uterine parameter typically leads to irregular spacing and clustering of an entire litter (Lu et al., 2013; Shi et al., 2014; Flores et al., 2020). For example, excessive intrauterine fluid results in crowding of implantation sites likely due to the disruption of physical and chemical interactions between the uterine epithelium and blastocysts (Lu et al., 2013). However, twinning is infrequently observed in this model (Lu et al., 2013). Since the frequency of twinning within and between *Mtrr* ^*gt/gt*^ litters was low and the general spacing of *Mtrr* ^*gt/gt*^ litters was otherwise normal, we propose an embryo-specific defect rather than a uterine-specific defect. Embryo-specific factors that affect uterine spacing are largely unknown (Chen et al., 2013). It is hypothesized that signals from a blastocyst (e.g., estrogen, prostaglandin, ephrin A) might initiate or maintain uterine events/molecular profiles required for optimal blastocyst spacing and implantation (Chen et al., 2013; Fujiwara et al., 2020). However, further experiments are required to better understand whether inequitable disruption of signaling among embryos of one litter can lead to poor spacing at a localized level (e.g., twinning). Additionally, it is unclear whether blastocysts secrete/receive inhibitory signals to/from neighboring blastocysts to maintain optimal uterine distance for fetal growth. Notably, other models of conceptus misalignment display extensive embryo spacing defects (Daikoku et al., 2011; Cha et al., 2014), yet it is unclear whether disrupted blastocyst signaling and/or poor responsiveness of the blastocyst to maternal signals is a common mechanism between twinning and orientation phenotypes.

The establishment of conceptus orientation is highly prescribed and depends on mesometrial-anti-mesometrial orientation in the uterus, ICM orientation within the blastocyst, and directional growth of the conceptus within the decidua (Chen et al., 2013). While the cellular and molecular events involved in embryo positioning largely remain unknown (Chen et al., 2013), factors intrinsic to the uterus are best studied. For instance, aberrant formation of uterine crypts misorient the blastocyst at implantation (Daikoku et al., 2011; Cha et al., 2014; Zhang et al., 2014), decidualization defects associate with misalignment of conceptuses post-implantation (Alexander et al., 1996; Zhang et al., 2014), and disorganization of the decidual vasculature network and increased blood perfusion misdirects trophoblast invasion resulting in skewed growth (Winterhager et al., 2013). The establishment of signaling gradients in decidua (e.g., WNT5) (Daikoku et al., 2011; Cha et al., 2014; Goad et al., 2017) and the responsiveness of the conceptus to these signals are also important for conceptus alignment. We observed low *Bmp2* mRNA expression in implantation sites of litters known to display conceptus skewing. However, a causative relationship was not established and it is unclear whether low *Bmp2* transcripts translate into low BMP2 protein levels or signaling activity. Further experiments are required to better understand the significance of this finding in the context of conceptus misalignment.

While the uterine-specific mechanisms indicated above are yet-to-be explored in detail in the *Mtrr* ^*gt*^ model, evidence presented here argues against a uterine defect as the main cause of conceptus misalignment. For instance, normal morphology and patterning of *Mtrr* ^*gt/gt*^ decidua was observed, and skewed F2 *Mtrr* ^*+/+*^ conceptuses were present after embryo transfer into a control uterus. As performed, the embryo transfer experiment ruled out an effect of the peri- and/or post-implantation stage uterus on conceptus alignment. However, based on the timing of the transfer into the recipient uterus, the F2 *Mtrr* ^*+/+*^ embryos might be exposed to abnormal factors in the oviductal/uterine fluid of the donor F1 *Mtrr* ^*+/+*^ females that might cause incorrect orientation at and after implantation. These factors are currently unknown but might be hormonal (Chen et al., 2013) and the effects would need to be long lasting and/or irreversible. To more definitively eliminate a uterine effect as a causative factor in embryo misalignment, additional embryo transfer experiments should be performed: *1*) The transfer of C57Bl/6J embryos (at E3.25) into an F1 *Mtrr* ^*+/+*^ uterus or *Mtrr* ^*gt/gt*^ uterus. Normal conceptus alignment would indicate no involvement of the peri- and/or post-implantation uterine environment. *2*) The transfer of F2 *Mtrr* ^*+/+*^ embryos at an earlier developmental stage (e.g., two-cell stage) or after *in vitro* fertilization into a control uterus. In this case, the effects of the oviductal fluid on conceptus alignment would be taken into account.

As an alternative to a defective uterine environment, a mechanistic defect intrinsic to the embryo might cause conceptus misalignment in the *Mtrr* ^*gt*^ model. Under normal circumstances, mouse blastocysts attach to the uterine epithelium *via* adhesion molecules (e.g., integrins, L-selectins) expressed on the mural trophectoderm (Cross et al., 1994; Red-Horse et al., 2004) so that the embryonic-abembryonic axis is parallel to the mesometrial-antimesometrial axis (Smith, 1985). How the blastocyst attaches to the uterine epithelium to establish a correct orientation is not well understood. One hypothesis is that the blastocyst might attach in any orientation and then roll along the uterine epithelium into the correct orientation (Kirby et al., 1967; Red-Horse et al., 2004). Otherwise, it was proposed that ICM cells might mobilise within the blastocyst to properly align in the implantation site after trophectoderm adhesion to the uterine epithelium (Kirby et al., 1967; Meilhac et al., 2009; Morris et al., 2010; Chen et al., 2013). Therefore, critical adhesion molecules on the outer membrane of the mural trophectoderm, the inner membrane of the trophectoderm, or the ICM of *Mtrr* blastocysts should be examined for regional expansion or misexpression as a cause for skewed growth. The occurrence of normal litter sizes from *Mtrr* ^*gt/gt*^ intercrosses at midgestation (this study; Padmanabhan et al., 2013) suggests that implantation of *Mtrr* ^*gt/gt*^ blastocysts is achieved regardless of this hypothetical adhesion defect. Alternatively, normal blastocyst orientation at implantation might be followed by unresponsiveness of some *Mtrr* conceptuses to cytokine signals from the decidua (e.g., WNTs, FGFs, BMPs) that guide mesometrial-antimesometrial alignment (Red-Horse et al., 2004; Daikoku et al., 2011; Cha et al., 2014; Goad et al., 2017). Further studies are required to elucidate the specific embryocentric mechanisms and molecular pathways involved in conceptus orientation, and how they are influenced by abnormal folate metabolism. Since MTRR enzyme is required for the transmission of methyl groups for methylation reactions in the cell (Shane and Stokstad, 1985), it is possible that epigenetic regulation of crucial genes required for conceptus orientation is disrupted in skewed *Mtrr* ^*gt/gt*^ conceptuses. Candidate genomic regions will be revealed by future whole methylome and transcriptome analyses in early embryos and uterine tissue.

It is unclear whether the phenotypes that underlie genetic disruption of abnormal folate metabolism (e.g., the *Mtrr* ^*gt*^ allele) are similar to maternal dietary folate deficiency, and what mechanistic parallels can be drawn between these two models. Although *Mtrr* ^*gt/gt*^ mice recapitulate developmental phenotypes (e.g., neural tube defects) and anemia observed in humans with folate deficiency (Padmanabhan et al., 2013; Ducker and Rabinowitz, 2017; Padmanabhan et al., 2018), diet-induced folate-deficient mice do not unless they are challenged with genetic insufficiency or viral infection (Heid et al., 1992; Koury and Horne, 1994; Burgoon et al., 2002; Burren et al., 2008). Uterine structure at implantation and the frequency of blastocyst implantation are likely unaffected by pre-conceptual dietary folate deficiency in female mice (Gao et al., 2012), though post-implantation decidualization and deciduoma formation might be inhibited (Geng et al., 2015). Decidualization was normal in *Mtrr* ^*gt/gt*^ mice at E6.5 (this study). Conceptus misalignment and twinning are yet-to-be reported in studies of folate deficiency or in other genetic mouse models where folate uptake or metabolism is disrupted leading to embryonic defects or lethality in early to mid-gestation (Piedrahita et al., 1999; Swanson et al., 2001; Gelineau-van Waes et al., 2008; Pickell et al., 2009; Salojin et al., 2011). Therefore, it is currently unclear whether these phenotypes are specific to the *Mtrr* ^*gt*^ model or a more general disruption of folate uptake/metabolism. Additional experiments should be performed to determine whether dietary supplementation of *Mtrr* ^*gt/gt*^ mice prevents the developmental phenotypes and/or conceptus misalignment observed.

Importantly, severe conceptus skewing at E10.5 in the *Mtrr* ^*gt*^ model exhibits a pattern of transgenerational inheritance. This non-conventional mode of inheritance is typically caused by exposure to an environmental stressor or metabolic disruption only in the initiating F0 generation and occurs independent of DNA base-sequence mutations (Blake and Watson, 2016). The *Mtrr* ^*gt*^ mouse line is a known model of transgenerational epigenetic inheritance as several embryonic phenotypes are inherited by the wildtype grandprogeny and great grandprogeny when either maternal grandparent is a carrier for the *Mtrr* ^*gt*^ mutation (Padmanabhan et al., 2013; Blake et al., 2021). While the mechanism of epigenetic inheritance of phenotypes is unknown in the *Mtrr* ^*gt*^ model and other mammalian multigenerational models, it is particularly difficult to explore when the phenomenon occurs *via* the maternal lineage. This is because defects in the maternal physiology and uterine environment are difficult to tease apart from abnormal germ cell factors (Watson and Rakoczy, 2016). For this reason, we utilized embryo transfer experiments to eliminate potential mechanistic effects of the peri/post-implantation uterine environment.

While an epigenetic mechanism is the most likely cause of this transgenerational effect, folate metabolism is required for cellular methylation and thymidine synthesis (Ducker and Rabinowitz, 2017). Therefore, disruption of folate metabolism potentially implicates both epigenetic and genetic instability. Notably, thymidine synthesis is likely unaffected by the *Mtrr* ^*gt*^ mutation since *de novo* mutations, which might transpire from low thymidine levels resulting in uracil misincorporation into DNA (Blount et al., 1997), occur at a similar frequency in C57Bl/6J control and *Mtrr* ^*gt/gt*^ mice (Blake et al., 2021). Therefore, we conclude that the wide range of phenotypes observed over multiple generations in the *Mtrr* ^*gt*^ model is not the result of genetic instability. Alternatively, considerable alteration in patterns of DNA methylation was identified in adult germ cells and somatic tissues of the *Mtrr* ^*gt*^ model, with some methylation changes associated with gene misexpression (Padmanabhan et al., 2013; Bertozzi et al., 2021; Blake et al., 2021). The range of developmental phenotypes observed in the *Mtrr* ^*gt*^ model (this study, Padmanabhan et al., 2013) suggests that this epigenetic instability occurs stochastically in each affected individual, ultimately leading to disruption of different genetic pathways and different phenotypes (Blake and Watson, 2016). This proposed stochasticity of inter-individual epigenetic changes might explain incomplete penetrance of the conceptus misorientation (and other phenotypes).

How abnormal epigenetic patterns are inherited is not well understood since they must circumvent reprogramming that occurs between generations or be reconstructed in the next generation (Jablonka, 2013; Blake and Watson, 2016). Altered patterns of DNA methylation and histone modifications, or misexpression of small non-coding RNA in germ cells are proposed candidates of epigenetic inheritance (Chen et al., 2016; Sharma et al., 2016; Blake et al., 2021; Lismer et al., 2021) and are currently being explored in the *Mtrr* ^*gt*^ model and other models of transgenerational epigenetic inheritance. While there is no evidence of abnormal mitochondrial function in *Mtrr* ^*gt/gt*^ livers (Sowton et al., 2020) or of small nucleotide polymorphisms in genes important for mitochondrial function in *Mtrr* ^*gt/gt*^ embryos with congenital malformations (Blake et al., 2021), oocyte mitochondria from F0 *Mtrr* ^*+/gt*^ or F1 *Mtrr* ^*+/+*^ females are yet-to-be assessed for defects. Notably, we discerned a greater frequency and later resolution of conceptus misalignment in wildtype conceptuses derived from an *Mtrr* ^*+/gt*^ maternal grandmother versus an *Mtrr* ^*+/gt*^ maternal grandfather. This observation might hold clues for future mechanistic exploration including the identification of different initiating factors in an F0 oocyte versus an F0 spermatozoon.

The association between folate metabolism and conceptus alignment has implications for early pregnancy loss associated with folate deficiency in humans, and for culture conditions involved in assisted reproductive technologies. Our study reinforces the importance of folate in healthy pregnancies, yet emphasizes that the role of folate metabolism during development is more complex than a direct uterine effect. Pre-conceptual supplementation of folic acid should be considered. Since conceptus skewing occurred several wildtype generations after the initial metabolic disruption, it is possible that unexplained pregnancy loss in humans might be associated with folate deficiency in the grandparental generation. This conclusion emphasizes the importance of folate fortification programs, which might take more than one generation to show the full effects.

## Data Availability

The original contributions presented in the study are included in the article/Supplementary Material, further inquiries can be directed to the corresponding authors.
