# Supplementary material for: Disruption of Folate Metabolism Causes Poor Alignment and Spacing of Mouse Conceptuses for Multiple Generations
Source: Front Cell Dev Biol. 2021 Dec 10;9:723978. doi: 10.3389/fcell.2021.723978 (PMC8703036; doi:10.3389/fcell.2021.723978)
Supplement: Supplementary file 1 [file DataSheet1.docx]

Supplementary Material

# Supplementary Figures and Tables

## Supplementary Tables

**Supplementary Table 1.** *In situ* hybridization probe primers and RT-qPCR primers

| **Gene name** | **Forward (5’-3’)** | **Reverse (5’-3’)** | **Reference** |
| --- | --- | --- | --- |
| *In situ* hybridisation probe primers | | | |
| *Tpbpa* | GTGAAGGCAAATTGTTAGGTGG | GGCAGTTAATTTGGGAGAGAGA | (Lein et al., 2006) |
| *Gcm1* | GCATCTACAGCTCGGACGACA | GGCCTTCCTCTGTGGAGCAGTC | - |
| *Syna* | TTGCAATCACACCTTTCAGC | TGGTGTCCACAGACAGGGTA | (Simmons et al., 2008) |
| *Hand1* | ATCATCACCATCATCACCACTC | CCCTTTAATCCTCTTCTCGCC | (Lein et al., 2006) |
| *Prl7b1* | GGGAGGACGTGGTCTCTGTA | CTTGCAAGGATAGCAAAGGG | (Lein et al., 2006) |
| T7 | TAATACGACTCACTATA | - | (Outhwaite et al., 2019) |
| T3 | AATTAACCCTCACTAAAG | - | (Outhwaite et al., 2019) |
| RT-qPCR primers | | | |
| *Bmp2* | CCTGAAGCAGAGACCCACCC | CTGGAAGTTCCTCCACGGCT | (Woods et al., 2017) |
| *Nr2f2* | GCCAGTACTGCCGCCTCAA | CAAACTGCCCGTGGGTAGGC | (Woods et al., 2017) |
| *Pgr* | GCCTATACCGATCTCCCTG | TTCCCTATGAGTGGCTTCTAC | (Li et al., 2017) |
| *Hand2* | CACCAGCTACATCGCCTACC | TCTCATTCAGCTCTTTCTTCCTCT | - |
| *Hoxa10* | CACAGGCCACTTCGTGTTCTT | TTGTCCGCAGCATCGTAGAG | - |
| *Mtrr* (total) | GGTTTTCCGCAGATCTTCAC | CTGTGTCAGGTGGGTCTCCT | (Padmanabhan et al., 2013) |
| *Mtrr* (wildtype) | GGGAAATTTGGAGCTATGTGG | CAGATGAGTCAAGACCCCAGT | (Padmanabhan et al., 2013) |
| *Polr2a* | GAGTCCAGAACGAGTGCATGA | ACAGGCAACACTGTGACAATC | (Solano et al., 2016) |

## Supplementary Figures


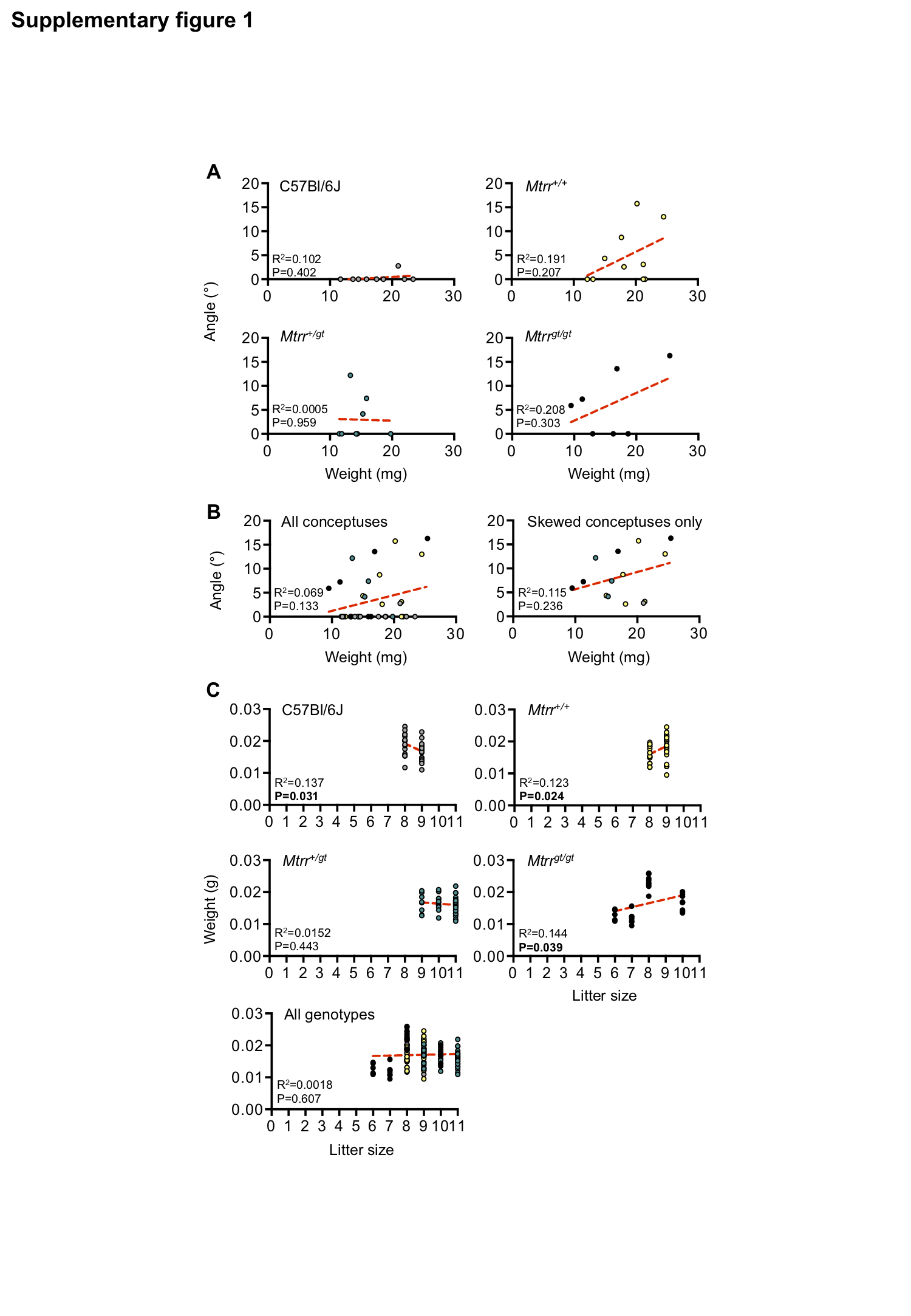


**Supplementary Figure 1.** **No correlation between *Mtrr^gt/gt^* implantation site weight and degree of skewing or litter size at E6.5.** (A-B) Linear regression analyses between whole implantation site weight and angle of conceptus relative to the central midline. (A) Weights and angles were measured in conceptuses derived from C57Bl/6J (grey dots), *Mtrr^+/+^* (yellow dots)*,* *Mtrr^+/gt^* (green dots), and *Mtrr^gt/gt^* (black dots) females mated with C57Bl/6J males. The maternal genotype is shown. (B) The data from (A) was pooled together (all conceptuses, left-hand graph) to increase sample size. Similarly, the data from the misaligned conceptuses (>2 s.d. above the mean conceptus angle in C57Bl/6J conceptuses, see Fig. 5B) were extracted and plotted to determine whether there was a correlation between implantation site weight and degree of misalignment. Red line indicates line of best fit. (C) Linear regression analyses of litter size and implantation site weight at E6.5 in conceptuses from C57Bl/6J (grey dots), *Mtrr^+/+^* (yellow dots)*,* *Mtrr^+/gt^* (green dots), and *Mtrr^gt/gt^* (black dots) females mated with C57Bl/6J males. The maternal genotype is shown. Red line indicates line of best fit.


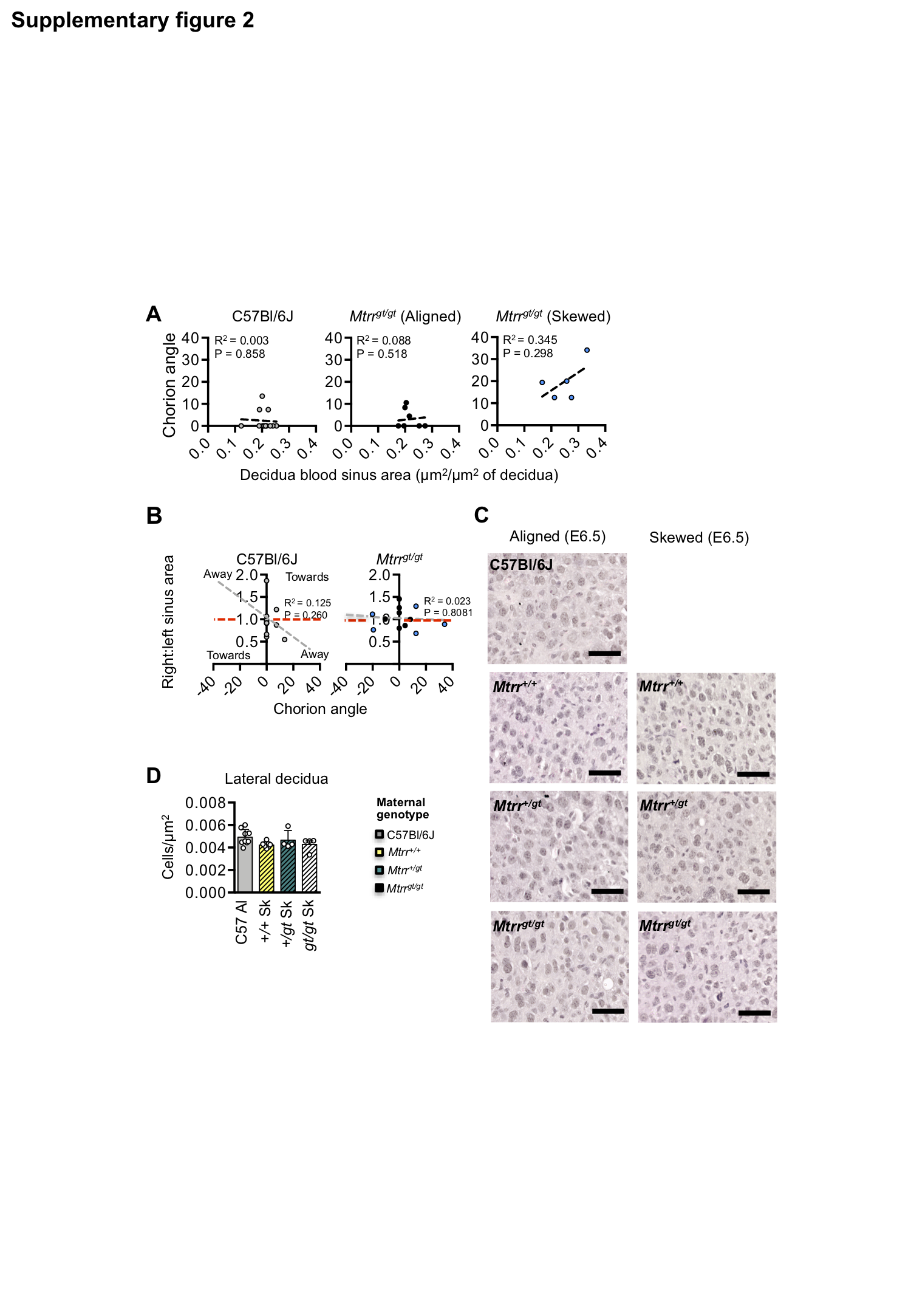


**Supplementary figure 2. Normal decidual blood spaces, decidual cell morphology and density regardless of *Mtrr^gt^* maternal genotype.** (A) Linear regression analyses of decidua blood sinus area in relation to chorion angle for C57Bl/6J (white circles) and *Mtrr^gt/gt^* conceptuses at E8.5. Aligned (black circles) and skewed (blue circles) *Mtrr^gt/gt^* placentas were considered separately (N=6-12 conceptuses/group). Dotted line indicates line of best fit. See also Figure 5E. (B) Linear regression analyses between the chorion angle and the ratio of right:left decidua blood sinus area in C57Bl/6J and *Mtrr^gt/gt^* conceptuses at E8.5 (N=6-12 conceptuses/group). Aligned (grey or black dots) and skewed (blue dots) conceptuses are shown. Dashed red line, right:left blood sinus area ratio of 1. Dashed grey line, line of best fit. See also Figure 5G. (C) Histological sections stained with H&E showing lateral decidua in implantation sites at E6.5 derived from C57Bl/6, *Mtrr^+/+^*, *Mtrr^+/gt^*, and *Mtrr^gt/gt^* females and C57Bl/6J males. The maternal genotype is indicated. Decidua associated with conceptuses that were aligned to or skewed from the midline is shown. Scale bars: 50 μm. (D) Graph showing the average number of lateral decidual cells per μm^2^ in histological sections from C57Bl/6 (grey bar), *Mtrr^+/+^* (yellow bar), *Mtrr^+/gt^* (green bar), and *Mtrr^gt/gt^* (black bar) females and C57Bl/6J males. Data is shown as mean ± sd. N=4-8 implantation sites/group with at least three histological sections assessed per individual. Al, aligned; Sk, skewed. One-way ANOVA.

# Supplementary References

Lein, E. S., Hawrylycz, M. J., Ao, N., Ayres, M., Bensinger, A., Bernard, A., et al*.* (2007). Genome-wide atlas of gene expression in the adult mouse brain. *Nature* 445, 168–176. doi.org/10.1038/nature05453

Li, R., Wu, J., He, J., Wang, Y., Liu, X., Chen, X., et al. (2017) Mice endometrium receptivity in early pregnancy is impaired by maternal hyperinsulinemia. *Mol. Med. Rep.* 15(5): 2503-2510. doi: 10.3892/mmr.2017.6322

Outhwaite, J. E., Patel, J., and Simmons, D. G. (2019). Secondary placental defects in *Cxadr* mutant mice. *Front. Physiol.* 10:622. doi: 10.3389/fphys.2019.00622

Padmanabhan, N., Jia, D., Geary-Joo, C., Wu, X., Ferguson-Smith, A. C., Fung E., et al. (2013). Mutation in folate metabolism causes epigenetic instability and transgenerational effects on development. *Cell* 155(1): 81-93. doi: 10.1016/j.cell.2013.09.002

Simmons, D. G., Natale, D. R., Begay, V., Hughes, M., Leutz, A., and Cross, J. C. (2008). Early patterning of the chorion leads to the trilaminar trophoblast cell structure in the placental labyrinth. *Development* 135(12): 2083-2091. doi: 10.1242/dev.020099

Solano, M. E., Thiele, K., Kowal, M. K., and Arck, P. C. (2016) Identification of suitable reference genes in the mouse placenta. *Placenta* 39, 7-15. doi:10.1016/j.placenta.2015.12.017

Woods, L. Perez-Garcia, V., Kieckbusch, J., Wang, X., DeMayo, F., Colucci, F., and Hemberger, M. (2017). Decidualisation and placentation defects are a major cause of age-related reproductive decline. *Nat. Commun.* 8(1):352. doi: 10.1038/s41467-017-00308-x
